# Evolution and Ecology of Silent Flight in Owls and Other Flying Vertebrates

**DOI:** 10.1093/iob/obaa001

**Published:** 2020-01-20

**Authors:** Christopher J Clark, Krista LePiane, Lori Liu

**Affiliations:** Department of Evolution, Ecology, and Organismal Biology, University of California—Riverside, 900 University Avenue, Riverside, CA 92521, USA

## Abstract

We raise and explore possible answers to three questions about the evolution and ecology of silent flight of owls: (1) do owls fly silently for stealth, or is it to reduce self-masking? Current evidence slightly favors the self-masking hypothesis, but this question remains unsettled. (2) Two of the derived wing features that apparently evolved to suppress flight sound are the vane fringes and dorsal velvet of owl wing feathers. Do these two features suppress aerodynamic noise (sounds generated by airflow), or do they instead reduce structural noise, such as frictional sounds of feathers rubbing during flight? The aerodynamic noise hypothesis lacks empirical support. Several lines of evidence instead support the hypothesis that the velvet and fringe reduce frictional sound, including: the anatomical location of the fringe and velvet, which is best developed in wing and tail regions prone to rubbing, rather than in areas exposed to airflow; the acoustic signature of rubbing, which is broadband and includes ultrasound, is present in the flight of other birds but not owls; and the apparent relationship between the velvet and friction barbules found on the remiges of other birds. (3) Have other animals also evolved silent flight? Wing features in nightbirds (nocturnal members of Caprimulgiformes) suggest that they may have independently evolved to fly in relative silence, as have more than one diurnal hawk (Accipitriformes). We hypothesize that bird flight is noisy because wing feathers are intrinsically predisposed to rub and make frictional noise. This hypothesis suggests a new perspective: rather than regarding owls as silent, perhaps it is bird flight that is loud. This implies that bats may be an overlooked model for silent flight. Owl flight may not be the best (and certainly, not the only) model for “bio-inspiration” of silent flight.

## Resumen

Proponemos y exploramos posibles respuestas a tres preguntas sobre la evolución y ecología del vuelo silencioso en lechuzas: (1) ¿Las lechuzas vuelan silenciosamente por sigilo o para reducir el auto-enmascaramiento?. La evidencia actual favorece levemente la hipótesis del auto-enmascaramiento, pero éste tema permanece irresuelto. (2) Dos de las características derivadas de las alas que aparentemente evolucionaron para suprimir el sonido del vuelo son los flecos del vexilo y la felpa dorsal de las alas de las lechuzas. Estas características ¿suprimen el ruido aerodinámico (sonido generado por el flujo de aire) o reducen en cambio el ruido estructural, tal como el ruido friccional de las plumas frotándose durante el vuelo? La hipótesis del ruido aerodinámico carece de apoyo empírico. Por el contrario, varias líneas de evidencia apoyan la hipótesis de que la felpa y el fleco reducen los sonidos friccionales, incluyendo: la posición anatómica del fleco y felpa, esta última mejor desarrollada en regiones del ala y cola propensos a frotación, y no tanto en áreas expuestas a flujo de aire ; la signatura acústica de frotación, que es de banda ancha e incluye ultrasonido, está presente en el vuelo de otras aves pero no en lechuzas; y la aparente relación entre la felpa y las bárbulas de fricción presentes en las remiges de otras aves. (3) ¿Evolucionó el vuelo silencioso en otros animales? Las características de las alas de las aves nocturnas (miembros nocturnos de Caprimulgiformes) sugieren que podrían haber evolucionado independientemente para volar de forma relativamente silenciosa, tal como ocurre en más de un gavilán diurno (Accipitriformes). Hipotetizamos que el vuelo de las aves es ruidoso porque las plumas alares están intrínsecamente predispuestas a frotarse y producir ruido friccional. La hipótesis sugiere una nueva perspectiva; en vez de considerar a las lechuzas como silenciosas, tal vez es que el vuelo de las aves es ruidoso. Esto implica que los murciélagos podrían representar un modelo ignorado de vuelo silencioso. El vuelo de las lechuzas podría no ser el mejor (y ciertamente no el único) modelo para la "bio-inspiración" del vuelo silencioso.

Palabras clave: Acústica, Aeroacústica, sonidos inducidos por locomoción, Strigiformes, ala

## Introduction

Owls are famous for their relatively silent flight. Most birds produce an audible signature with every flap of their wings, but the wing noise of many (though not all) owls is low enough that a human often does not hear an owl flying by, even at close range. Why have they evolved to do this? The answer is not entirely clear. Here we explore answers to three intertwined questions about the evolution of silent flight. The questions are: (1) do owls fly silently to reduce self-masking, or for stealth during an ambush? (2) Do wing features that promote silent flight do so by suppressing aerodynamic sound, or by suppressing structural sound, such as made by friction between feathers during flapping? And, (3) has silent flight convergently evolved in flying animals with ecological similarities to owls, such as nightbirds (nocturnal members of Caprimulgiformes), or hawks? Research on this topic has focused narrowly on owls, and many works imply that owls are unique or the only birds to have evolved silent flight (Kroeger et al. 1972; Lilley 1998). Might other flying taxa such as harriers or nightbirds also offer simple, general lessons about silent flight?

Our purpose in asking these questions is two-fold. First, we review what is known about the evolution and ecology of silent flight, hunting, and nocturnality. Two disparate fields have extensively studied questions on either side of this topic: Engineers have studied the physical acoustics of owl flight, most often in a simple aerodynamic context such as a dried, spread owl wing placed in a wind tunnel, intended to simulate sounds of gliding flight (e.g., Kroeger et al. 1972). Sensory neurobiologists have studied owl hearing, mainly in Barn Owls (*Tyto alba*), as a model system of how the brain localizes sound (e.g., Konishi 1973a; Knudsen and Konishi 1979). In between the ground covered by these two fields is the sensory and evolutionary ecology of sound in owl hunting. Here, we explore this space in between aerodynamics and neurobiology. We cover both how and why owls fly silently, since, in functional morphology, how a trait works is linked to why it has evolved. Our purpose is to point out tractable questions about silent flight that a biologist should be able to answer. Second, we examine a few of the assumptions about owl flight that are currently widespread in the literature.

Some of the terminology and ideas that we revisit have their origin in Graham’s (1934) foundational paper, “The silent flight of owls.” This is the landmark paper on this topic: Graham appears to have been the first to establish the owl wing morphologies associated with the acoustics of owl flight. While most of his ideas about mechanism have stood the test of time, a few are wrong or oversimplified. For instance, his term “trailing edge fringe” has misled subsequent authors for reasons we describe below.

Current owl flight literature implies the mechanisms by which owl wings reduce noise in flight are aerodynamic mechanisms, caused by noise made by air flowing over the wing. For example, a recent review of owl wing features focused almost exclusively on postulated aerodynamic mechanisms (Wagner et al. 2017), and scarcely mentioned that there is another explanation for two wing features, the velvet and the vane fringes, given in books on raptor biology (e.g., Duncan 2003; Peeters 2007) or feather anatomy (Lucas and Stettenheim 1972). Below we develop this alternative and the empirical support for it, a version of which was originally proposed by Graham (1934), which we call the structural noise hypothesis. The central idea is that feathers make substantial frictional sound when they rub against each other (Lucas and Stettenheim 1972) and impact sounds during other physical interactions such as in impacts against other structures such as dry grass.

### Owl diversity and phylogeny

**Fig. 1:**
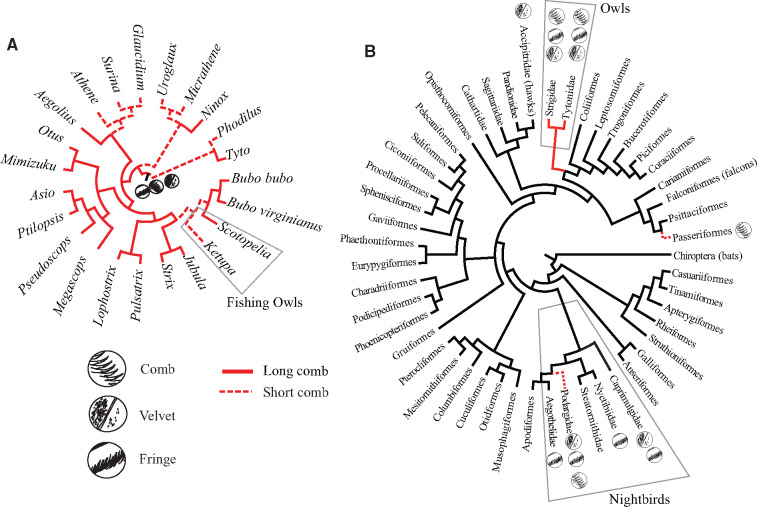
Phylogenetic distribution of dorsal velvet, fringed vanes, and leading-edge comb. **A**) Phylogeny of major owl genera (modified from Wink et al. 2009). Red: leading-edge comb extends >1 mm from surface of P10 (as measured at the midpoint of the comb) in at least one species within each clade. Red dash: leading-edge comb present but extends <1 mm at midpoint of the comb. **B**) within extant flying vertebrate clades (phylogeny from Prum et al. 2015). Clades with at least one species with a character are marked (i.e., out of ∼5000 passerine species, since the two *Stelgidopteryx* spp. have a comb-like structure, the entire clade is indicated as short comb present). Black: no data; true absence data are lacking.

Owls (clade Strigiformes) are a natural (monophyletic) group (Fig. 1A). Owls have two major subclades, the barn owls (Tytonidae, ∼20 spp.) and “true” owls (Strigidae, ∼200 spp.) (Uva et al. 2018). Tytonids are medium to large (187–1260 g), and generally have a mammalian diet (Bruce 1999). Strigids vary by two orders of magnitude in size, from the 40 g Elf Owl (*Micrathene whitneyi*) (Holt et al. 1999a) to the Eurasian Eagle Owl (*Bubo bubo*), which can weigh up to 4 kg (Holt et al. 1999b). All owls are predators of other animals, which they hunt on the wing. Small owl species tend to eat insects, whereas large species eat mammals, fish, birds, or a mix. Most are nocturnal or crepuscular, and a few are diurnal. This diurnal/nocturnal divide does not always cleanly separate owls into visually hunting versus acoustically hunting species. Species hunting in daylight, such as Snowy (*Bubo scandiacus*) or Great Gray Owl (*Strix nebulosa*) may nevertheless be entirely reliant on acoustic cues to detect prey, when they hunt prey under a layer of snow or other visual barrier (Chamberlin 1980). Hearing is acute in some owl species, and many species have specializations associated with hearing, such as an enlarged facial disc and asymmetrical ears (section “Facial disc”). Owls have evolved specialized wing features that are related to sound production while hunting (section “How do owls have silent flight?”).

Within birds, owls are sister to a large, ecologically diverse clade, Coraciimorphae (Prum et al. 2015; Fig. 1B). This phylogenetic location offers a couple considerations for outgroup comparisons. Owls are not closely related to either of the major clades of diurnal raptors, hawks (Accipitriformes) or falcons (Falconiformes). Owls have independently evolved ecological similarities to these other groups, such as carnivory, large body size, and aerial pursuit of prey. Another group to which they have ecological affinities but are distantly related is a phylogenetic grade of nocturnal birds within Caprimulgiformes (nightjars, nighthawks, frogmouths, but not including the diurnal Apodiformes, which are phylogenetically nested inside Caprimulgiformes). We hereafter refer to this grade as the Nightbirds (Fig. 1B). Trait owls convergently share with Nightbirds include nocturnality, aerial hunting, and apparently, silent flight. What these evolutionary relationships suggest is that phylogenetic comparison with diurnal raptors, nightbirds, or other outgroups will be appropriate, depending on the question(s) under investigation. Several implications raised in the “Do other flying animals have silent flight?” section, such as questions about the acoustics of bat flight (which is virtually unstudied), arise directly out of this phylogenetic perspective.

Barn Owls have proven a tractable study system and so a substantial amount of information about them is available, especially about their neurobiology of hearing. However, as Tytonids are somewhat distantly related to the more speciose strigid owls, whether patterns demonstrated for Barn Owls specifically are general to owls as a whole is at times unclear. Since certain anatomical aspects of the wings have been carefully described for Barn Owls (Bachmann et al. 2007) but not a strigid, here we include a small amount of data obtained from visual inspection of *N* = 10 specimens of a strigid, Barred Owl (*Strix varia*) we obtained from the US Forest Service (Wiens et al. 2019).

## Why do owls have silent flight?

What function does silent flight serve, and why has it evolved? Noises produced by owl wings are potentially audible to the owl itself, to prey, to other owls (conspecifics), and to parasites or predators. No prior work on owls has suggested that silent flight has evolved in response to selective pressures exerted by conspecifics, predators, or parasites. Therefore, we assume owls have evolved silent flight to aid in their own hunting, and not one of these other possible functions.

**Fig. 2:**
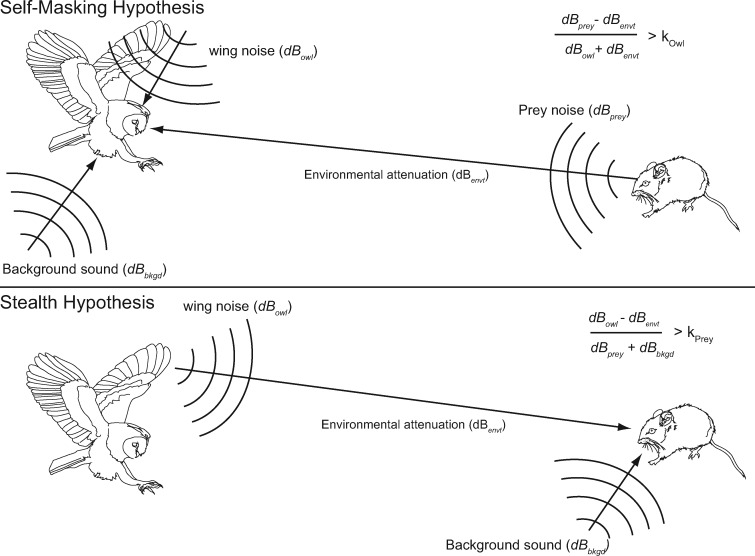
The self-masking hypothesis (**A**) and the stealth hypothesis (**B**) for the evolution of silent flight, expressed as a masking ratio: signal/masker>*k*, where *k*, hearing ability, is the ability of the owl (*k*_owl_) or prey (*k*_prey_) to discriminate signal from masker. **A**) According to the self-masking (“owl ear”) hypothesis, silent flight reduces the degree to which owl wing sounds mask their own hearing. Wing noises dB_owl_ are a masker, as is background (environmental) sound dB_bkgd_. The signal are prey sounds dB_prey_, such as rustling, chewing, or vocalizations that the owl uses to locate prey. dB_prey_ attenuates with distance and other sources of environmental transmission loss, dB_envt_. **B**) According to the stealth (“mouse ear”) hypothesis, silent flight reduces the ability of prey to hear the owl approach. In this model, the wing noises dB_owl_ are the signal that is attenuated by environmental effects (dB_envt_) such as distance or snow. Masking the sound of the approaching owl is dB_bkgd_, while the prey’s hearing ability (*k*_prey_) determines whether the prey hears the owl.

Regarding hunting, there are two hypotheses of function (Fig. 2). According to the self-masking hypothesis, owls fly silently to avoid producing wing noises that block their own hearing, much as the sound of one’s own footsteps can mask the ability to hear another noise (Moore 1989). According to this hypothesis, by reducing dB_owl_, owls can better locate the noises prey make. The other hypothesis is the stealth hypothesis: silence allows an owl to remain undetected by prey. Reducing the prey’s ability to hear the owl’s flight sound (dB_owl_) limits the prey’s ability to take appropriate evasive action to avoid the owl’s strike.

### The self-masking hypothesis

Masking is defined as the presence of one sound making another sound inaudible (Moore 1989). Masking can be simultaneous or non-simultaneous. In non-simultaneous (temporal) masking, the masker and signal are not present at the same time: a sudden, loud sound makes signals immediately before or after it inaudible (Moore 2007). In simultaneous masking, the masker and the signal are present at the same time (Moore 1989). A given signal frequency has a bandwidth of sounds that are best at masking it (Moore 1989). In Barn Owls, signals of 2, 4, 6.3, and 8 kHz have masking bandwidths of 81, 218, 562, and 831 Hz, respectively (Dyson et al. 1998). This means that for a signal of a tonal 2 kHz sound, noise played within an 81 Hz bandwidth of that signal will be an effective masker (Quine and Konishi 1974; Dyson et al. 1998).

According to this “owl ear” hypothesis, silent flight allows an owl to hear and localize prey better. There are five variables that influence self-masking (Fig. 2A): the sound produced by the owl’s wings (and body) in flight (dB_owl_), the sound produced by the prey (dB_prey_), the background sound of the environment (dB_bkgd_), the transmission loss of the prey sound through the environment to the owl (dB_envt_), and the hearing ability (*k*_owl_) of the owl to localize the prey sound in the presence of masking sounds. The transmission loss of the owl’s wing noise is essentially constant (since the distance between wing and ear is approximately invariant), and thus is not included as separate parameter. Rather, dB_owl_ is evaluated at the location of the owl’s ears, since it is only the wing noise that reaches the owl’s ears that blocks the owl’s hearing.

Expressing these variables as a simple masking (signal to noise) ratio in which the signal reaching the owl is (dB_prey_ − dB_envt_) while the masker is (dB_owl_ + dB_bkgd_) yields:

(1) $$({\text{dB}}_{\text{prey}}-{\text{dB}}_{\text{envt}})/({\text{dB}}_{\text{owl}}+{\text{dB}}_{\text{bkgd}})>k_{\text{owl}}.$$

Like all models, this is a simplification, each of these variables has a complex physical basis, which we review below. The suggestion that they can be added or subtracted is intended as a qualitative thought example; masking is more complex (see above) than is implied by this model.

This self-masking hypothesis predicts that silent flight evolved in response to how reliant the owl is on sound to hunt. This hypothesis predicts that wing features promoting silent flight will be correlated with traits associated with acute hearing (i.e., traits suggesting *k*_owl_ is good). It predicts that owls hunting prey that make audible noises (dB_prey_ is not negligible) which allow them to be localized will have these wing features, whereas those specializing on prey that do not make noises that allow the owl to hunt the prey acoustically will not have silencing features. This hypothesis predicts that the silencing features of owl wings will reduce self-noise within the range of frequencies that are best at masking sounds owls rely on most to localize prey.

#### Sound field shape

According to this hypothesis, the wing features are selected to reduce sound *above* the wing that is directed inward, toward the owl’s head. Moreover, wing noise is not strictly a far-field problem, since owl ears are within the near-field of the low end of the sound spectrum produced by the wings. This hypothesis does not predict that owl wing features would evolve to reduce noise projected below, above, or behind the owl specifically, since noise in these directions will tend to not reach the owl’s ears (although sound in these directions could be reduced as a byproduct of a feature that reduces sound radiated in multiple directions).

### The stealth hypothesis

Stealth is the ability to remain undetected by a target until it is too late for the target to evade an attack (Heithaus et al. 2002). According to this “mouse ear” hypothesis, silent flight allows an owl to sneak up on prey (e.g., mice). The five factors that influence the prey’s ability to hear the owl approach are (Fig. 2B): the amount of background sound in the environment (dB_bkgd_), the sound the prey itself makes (dB_prey_) from chewing, moving, or vocalizing, the sound produced by the owl’s wings (dB_owl_), the transmission loss of the owl wing noise through the environment to the prey (dB_envt_); and the prey’s ability to hear the owl, *k*_prey_. As in the self-masking hypothesis, the stealth hypothesis can be expressed as a masking ratio, where the signal to the prey is dB_owl_ − dB_envt_, and the masker is dB_bkgd_ + dB_prey_:

(2) $$({\text{dB}}_{\text{owl}}-{\text{dB}}_{\text{envt}})/({\text{dB}}_{\text{bkgd}}+{\text{dB}}_{\text{prey}})>k_{\text{prey}}.$$

As in Equation (1), this model is a simplification for the purposes of hypothesis generation.

The stealth hypothesis predicts that silent flight is not related to how good *owls* are at hearing, and instead evolves in response to *k*_prey_, which is the prey’s ability to hear the signal (dB_owl_ − dB_envt_) in the presence of background noise (dB_bkgd_ + dB_prey_). It predicts that owls hunting prey that use hearing to evade predators will have silencing features. The stealth hypothesis predicts that the silencing features that owls have are selected to reduce noise at all frequencies to which the prey may be sensitive (*k*_prey_).

#### Sound field shape

Prey are usually in front of an attacking owl, and are in the far-field up until the final moments of the strike. According to the stealth hypothesis, wing features reduce far-field sound projected *forward* from the owl. According to this hypothesis, sound shed behind, below, to the sides, or above the owl will not be selected to be reduced since the prey is not in these locations except in rare circumstances, e.g., an owl strikes but misses because the prey runs toward the owl, causing it to overshoot (Ilany and Eilam 2008). (As before, sounds in these directions could be reduced as a correlated byproduct of reducing sound projected in front of the owl).

Evaluating these models requires more information about how owls hunt, as the nature of dB_prey_, dB_envt_, dB_owl_, dB_bkgd_, *k*_owl_, and *k*_prey_, each has a complex physical basis.

## How owls hunt

Owls hunt using a combination of visual and acoustic cues; owls are not known to use other sensory modalities to localize prey (such as smell or touch). A few species seem to use primarily visual cues (e.g., fishing owls, pygmy owls) (Bündgen 1999; Holt and Petersen 2000), whereas some hunt using exclusively acoustic cues (such as owls hunting voles through a layer of snow), and others use varying degrees of both light and sound. For example, crepuscular owls hunt when light from the rising or setting sun is available, as well as sound (Peeters 2007). By contrast, fully nocturnal species have only limited ambient light available, particularly when moonlight is not available.

### Owl visual acuity

The low levels of light at night poses a visual challenge for nocturnal animals. Whereas a typical value of ambient light available for diurnal animals in direct sunlight is 129,000 lux (lumens m^−2^) and on an overcast day is 100–2,000 lux, light levels fall dramatically as the sun sets. Under a full moon, nocturnal light levels can be as high as 50–100 mlux, whereas under a new moon, light levels can be as low as 0.7–3 mlux (Hänel et al. 2018, table 2). To deal with low levels of ambient light, nocturnal owls have enlarged eyes, and their retinas contain primarily rods, with relatively few cones, to maximize reception of light (Walls 1942). In open environments, there may always be enough ambient light for owls to utilize visual information in hunting (Dice 1945; Martin 2017). However, in forest understory on moonless nights, light levels at the forest floor may frequently fall below levels necessary for spatial resolution (Martin 2017).

Despite adaptations for nighttime vision, no enhanced light-gathering capability of an owl’s eyes can overcome environments with minimal light. Since vision is one of the primary ways flying animals sense and avoid obstacles, nocturnal animals risk collisions with environmental objects when they fly. Bats frequently collide with environmental objects, for instance (Orbach and Fenton 2010). Strictly nocturnal owls hunting under the forest canopy may mitigate this by employing a sit and wait hunting strategy (Martin 2017). By utilizing a small number of perches in a small territory, owls may rely on spatial memory of their environment to avoid collisions with large objects (Martin 2017). Nevertheless, owls collide with environmental objects such as adhesive vegetation (Palmer et al. 2009; Rodríguez et al. 2009), or anthropogenic structures such as fences (Allen and Ramirez 1990), and electrical lines (Ii 2005). Longland and Price (1991) describe that Great Horned Owls occasionally collided with bushes when attacking rodents, and flew more quickly toward prey when the moon was full (flight speed of 8.4 ± 0.63 m s^−1^) than under a new moon (flight speed of 7.1 ± 0.96 m s^−1^), i.e., when visual cues about obstacles are least available. We hypothesize that brushing against or directly colliding with substrates is more common in nocturnal flying animals than in diurnal, and thus nocturnal animals may be selected to evolve features that mitigate the effects, including acoustic effects, of collisions.

### Noises owls hear (*k*_owl_)

Owls have more sensitive hearing than other birds (Dooling 1992). There is interspecific diversity in hearing ability (Table 1). Of owls tested, the African Wood Owl (*Strix woodfordii*) and Northern White-faced Owl (*Otus leucotis*) are the most sensitive to low frequency sounds, able to detect a 500 Hz sound at an intensity of −9.5 dB (van Dijk 1973; Nieboer and Paardt 1977). Audiograms indicate that the European Eagle Owl (*B. bubo*) has the highest high frequency cutoff (13.8 kHz). On average, owls are most sensitive to sounds from 2 to 4 kHz (Table 1).

**Table 1:** Hearing ability of various birds, including owls (adapted from table 1 in Dyson et al. 1998)

Species	*n*	Best frequency (kHz)	Audible intensity of best frequency (dB)	Low frequency sensitivity (dB)	High frequency cutoff	Reference
Passerines	13	2.9	5.1	32.4	9.7	Dooling (1992)
Non-passerines	8	2.1	8.5	27	7.5	Dooling (1992)
Strigiformes	2	2.7	−17.6	1	11.2	Dooling (1992)
*Tyto alba guttata*	3	6.3	−14.2	7.0	13.8^a^	Dyson et al. (1998)
*Tyto alba pratincola*	1	4	−18.6	4.8	12.9^a^	Konishi (1973)
*Asio otus*	6	6	−21.5	−6.5	11.1^a^	van Dijk (1973)
*Otus scops*	1	4	−6.0	−0.5	9.5^a^	van Dijk (1973)
*Otus leucotis*	1	2	−15.0	−9.5	9.3^a^	van Dijk (1973)
*Ketupa zeylonensis*	1	1	−9.0	7.5	–	van Dijk (1973)
*Bubo scandiacus*	1	4	−18.0	−8.	8.5^a^	van Dijk (1973)
*Bubo bubo*	1	2	−20.0	−1.5	8.6^a^	van Dijk (1973)
*Bubo virginianus*	1	1	−16.0	−1.6	7.0	Trainer (1946)
*Bubo nipalensis*	1	0.5	−5.0	−5.0	7.7^a^	van Dijk (1973)
*Strix virgate*	1	0.5	−7.5	−7.5	11.3^a^	van Dijk (1973)
*Strix seloputo*	1	2	−12.5	−7.5	9.4^a^	van Dijk (1973)
*Strix aluco*	6	6	−17.5	−1.0	10.3^a^	van Dijk (1973)
*Strix woodfordii*	1	6	−15.0	−9.5	10.0^a^	Nieboer and Paardt (1977)
*Aegolius acadicus*	16	4	4.3	–	8.6	Beatini et al. (2018)
*Megascops asio* ^b^	13	4–5.7	20^a^	60^a^	8^a^	Brittan-Powell et al. (2005)

^a^Data extrapolated from audiograms.

^b^From auditory brainstem response recordings, which can underestimate actual thresholds (Brittan-Powell et al. 2005).

Owls have the ability to accurately locate pure tones in three-dimensional space (Payne 1971a; Olsen et al. 1989). This remarkable ability varies with signal frequency (Payne 1971a; Olsen et al. 1989). Their ability to localize is made possible through the interaction between three morphological features: the facial disk, asymmetrical ears, and the organization of sound localization areas in the brain (Payne 1971a; Konishi 1973b; Olsen et al. 1989).

#### Facial disc

Many owls have a facial disc, a circular array of feathers under muscular control that gives owls their distinctive human-like face (Konishi 1973a; Norberg 1977). Sound arriving at an owl interacts first with the facial disc, which reflects and filters sound as it travels to the ear canal (Konishi 1973b). Species such as *Strix* spp., *Tyto* spp., and *Aegolis* spp. have large, well developed facial discs, whereas many other owls have small facial discs. Some fishing owls in the genus *Scotopelia* lack the facial disc entirely (Bündgen 1999; Stiles 1999). Harriers (*Circus* spp.) have convergently evolved a facial disc (Thiollay 1994). The facial disk primarily funnels sounds with wavelengths shorter than the diameter of the face, approximately >3 kHz for a Barn Owl (Knudsen and Konishi 1979; Knudsen 1981). When the facial ruff of a Barn Owl is removed, the bird maintains its ability to locate sounds in the horizontal plane (azimuth) but its ability to locate sounds in the vertical plane (elevation) is impaired (Hausmann et al. 2009). Their ability to detect elevation arises from an interaction between the facial disc and asymmetrical ears (Knudsen 1981). Most work on the facial disc has been done on Barn Owls (von Campenhausen and Wagner 2006); how variation in facial disc size (e.g., the enormous facial disc of Great Gray Owl, *S. nebulosa*) influences sound reception is not clear.

#### *k*_owl_: ear

After interacting with the facial disc, sound travels down the ear canal to the tympanic membrane. The ability of a Barn Owl to accurately locate prey depends on interaural cues, comparison of sounds arriving at each ear (Knudsen 1981). This comparison is aided by the morphological asymmetry of the ear canal of some owls, such as Barn owl, meaning the morphological shape of the left ear differs from that of the right (Norberg 1977). Owls use two aspects of incoming sound, the interaural level differences (ILDs) and interaural time differences (ITD), to locate a sound source (Payne 1971a; Knudsen and Konishi 1979; Knudsen 1981; Olsen et al. 1989). In many owls, the right ear is more sensitive to sounds coming from above the midline of the face while the left ear is more sensitive to sounds coming from below (Knudsen and Konishi 1979). Thus, the ILD allows owls to locate vertical source (elevation) of a sound (Olsen et al. 1989). The organization of ILDs in the Barn Owl’s brain varies with signal frequency (Olsen et al. 1989). At lower frequencies (<4 kHz), ILDs also vary with azimuth and have a low spatial resolution (Olsen et al. 1989). At higher frequencies (>5 kHz), ILDs vary primarily with elevation and have a high spatial resolution, resulting in a more precise auditory map (sound source location) (Olsen et al. 1989). Thus, sounds >3 kHz (and especially >5 kHz) help a Barn owl localize prey elevation.

For sound frequencies between 2 and 10 kHz, Barn Owls use ITDs to locate sounds in azimuth: sounds arriving at the left ear first are from the left side, and sounds arriving at the right ear first are from the right. For frequencies <4 kHz, ITDs vary with azimuth only (Olsen et al. 1989). At higher frequencies, ITD also varies with source elevation, but the organization of ITD variation with elevation in the Barn owl’s brain is much less systematic than ITDs with azimuth (Olsen et al. 1989). Thus, while a wide range of frequencies allows detection of ITD, the sounds that work best for localizing azimuth are frequencies in which the wavelength is greater than the diameter of the bird’s head, i.e., <3 kHz. Sounds that mask a Barn Owl’s ability to hear sounds <3 kHz are thus expected to impair an owl’s ability to use ITD cues to localize the azimuth of incoming sound.

In the Barn Owl’s brain, spatial information provided by ITDs and ILDs are combined, generating a single point source for the signal. While Barn Owls can accurately locate pure tones, strike accuracy increases as bandwidth (range of frequencies present in a signal) increases, particularly the inclusion of high frequencies (Knudsen 1981). Knudsen and Konishi (1979) found that Barn Owls are most accurate at localizing sounds between 4 and 8 kHz and are reluctant to respond to sounds outside of 3–8 kHz (Knudsen and Konishi 1979). Using a live bird trained to strike at pure tones, Konishi (1973b) found that Barn Owls were accurate when presented with pure tones between 3 and 9 kHz. Outside of this range, the strike error increased dramatically (Konishi 1973b). Thus, the components of sounds produced by a potential prey item in the range of 3–9 kHz are most useful to a Barn Owl.

Barn owls have been studied much more extensively than strigids. Not all strigid owls have asymmetrical ears, and of owls that do have asymmetrical ears, there are substantial differences between species, such as whether the asymmetry is in the skull or the fleshy part of the ear. One estimate suggests that asymmetrical ears have evolved five separate times in owls (Norberg 1977). Great Horned Owl (*Bubo virginianus*) have symmetrical ears, and ILD cues reinforce azimuth discrimination, rather than elevation (Volman and Konishi 1990; Beitel 1991). Volman and Konishi (1990) suggest that no species of owl with symmetrical ears hears well >6 kHz, whereas all asymmetrically-eared owls do. To what degree body size (e.g., interaural distance) affects hearing is not clear. Northern Saw-whet owls (*Aegolius acadicus*) are among the smallest owls with highly sensitive hearing and asymmetrical ears (Frost et al. 1989; Beatini et al. 2018). Since they are smaller, their interaural distance is slightly shorter than that of a barn owl, but to what degree this affects the frequencies that provide ILD versus ITD cues is not entirely clear.

According to the self-masking hypothesis, in owl species with asymmetrical ears, wing sounds >3 kHz (and especially, 5–9 kHz) are the likeliest sounds to mask the owl’s ability to use ILD cues to detect prey elevation; lower frequency wing sounds (1–3 kHz) are predicted to mask the owl’s ability to use ITD cues to detect prey azimuth. By contrast, according to the self-masking hypothesis, in species with symmetrical ears, wing sounds >5 kHz do not mask the owl’s hearing, and wing sounds from 1 to 5 kHz would mask the owl’s ability to discriminate prey azimuth. One way to test the self-masking hypothesis would be to experimentally manipulate wing features, causing an owl to make extra wing sound, then test the owl’s ability to localize prey sounds in the presence of its own wing sound. If such a manipulation masked an asymmetrically-eared owl’s hearing in the 3–9 kHz band, the prediction is it would tend to miss prey in elevation more so than azimuth, whereas if the manipulation masked hearing <3 kHz, the prediction is it would miss prey in azimuth more so than elevation. By contrast, the same manipulation performed on a symmetrically-eared owl would be predicted to have no effect on elevation discrimination.

### Noises that prey make (dB_prey_)

Acoustically hunting owls listen for and localize in space the sounds that prey makes. Self-masking is predicted to apply to owls that can use hearing to detect those prey that actually make sounds (dB_prey_ is not negligible). Mammals produce locomotion-induced sounds (Clark 2016) such as rustling when moving through dry grass or similar substrates. Rodents also produce gnawing or chewing noises. Arthropods similarly produce broadband, click-like locomotion-induced sounds when they walk on substrates (Goerlitz et al. 2008). Both chewing and locomotion-induced sounds tend to be broadband (Konishi 1973a; Goerlitz et al. 2008). Thus far, experiments on owl localization abilities have focused on sounds that resemble rodent rustling or chewing sounds (e.g., Konishi 1973a).

Many rodents also vocalize, some of which are audible to owls and others of which are ultrasound, i.e., at frequencies not audible to owls (Wöhr and Schwarting 2013). Other acoustically hunting predators and parasites, such as bats hunting frogs (Halfwerk et al. 2014) or flies hunting crickets (Pascoal et al. 2014) eavesdrop on and locate prey from prey communication sounds. We did not find any reports of owls localizing prey by eavesdropping on prey communication sounds.

Certain bats use locomotion-induced sounds (footsteps, rustling, etc.) of terrestrial arthropods such as of crickets and grasshoppers to passively locate and catch their prey on the ground (Bell 1982; Razak et al. 2007). We did not find any reports of insect-eating owls using a similar hunting strategy. Other prey types (e.g., birds) tend to be silent at night, thus owls apparently have less capacity to localize bird prey using sound.

According to the stealth model, dB_prey_ masks the prey’s ability to hear the owl. Therefore, owls should approach potential prey while that prey is making sound; and prey will better detect predators by producing sound intermittently, listening for owls during pauses of making sound when dB_prey_ is negligible. One common response of prey to a possible predator is to freeze in place, thereby ceasing to produce dB_prey_.

### Noises that prey can hear (*k*_Prey_)

Owls specialize on eating fish, arthropods, birds, mammals, or are generalists. While some owls include non-avian reptiles or amphibians in their diet, these species tend to be generalists. The hearing mechanisms of all of these potential preys are too diverse to review in detail here, but there are some general features. Fish hearing appears to be irrelevant; the much greater density of water causes airborne sound to reflect off of the air–water interface, meaning that a fish is unlikely to hear an airborne predator approaching even if it is noisy.

#### Arthropod hearing

Many clades of insects have independently evolved ears, which they use both for communication and predator detection, especially to avoid the echolocation calls of bats (Conner and Corcoran 2012; Fournier et al. 2013; Strauß and Stumpner 2015). Many insect ears are intrinsically most sensitive to ultrasound, because tympana tend to evolve from cuticle patches that are typically small and light, and thus have high resonance frequencies (Strauß and Stumpner 2015). Although some insects (e.g., moths) evolved ears at approximately the same time that bats evolved echolocation, roughly 60 million years ago, other insects such as Caeliferans (Orthoptera) evolved ears well before bats arose, and do not use hearing for communication. This implies Caeliferans evolved ears for detection of another predator, such as birds (reviewed in Strauß and Stumpner 2015).

Whether insects use hearing to detect predatory birds has received limited attention. Fournier et al. (2013) document that noctuid moths and butterflies could hear the atonal, broadband wing sounds of approaching passerine birds. Moreover, there is one report of insects evading bird wing noises in the wild: moths (*Helicoverpa armigera*) reacted to rustling sounds created by the wings of Cape Bulbuls (*Pycnonotus capensis*) moving through bushes (Jacobs et al. 2008). Rustling sounds caused by wing–substrate interactions are broadband and include ultrasound. Therefore, it is possible that flying insects could respond to dB_owl_ of an approaching predator, and insects are particularly likely to be sensitive to ultrasonic component of flight noise. There do not appear to be any examples of terrestrial insects or other arthropods (e.g., spiders, scorpions) responding to flight sound while on the ground, and eared insects that evolve flightlessness tend to also evolve to lose their ears (Strauß and Stumpner 2015), which suggests that hearing is of limited use as an anti-predator device to terrestrial arthropods. Thus, birds that catch insects through aerial hawking (i.e., taking flying insects while on the wing) could be selected to reduce wing sounds for stealth (especially of ultrasound), but there is no current evidence that birds catching terrestrial insects would be selected to reduce wing sounds for stealth.

#### Bird hearing

Birds tend to have good broad-spectrum hearing (Dooling and Prior 2017) and birds employ hearing extensively in anti-predator responses, such as by responding to alarm calls uttered by other birds. However, apart from nightbirds and owls, most birds are diurnal, and sleep at night (except during migration). We are unaware of any published examples in which birds listen or react to predator wing noises, especially when asleep. The few owls that are bird specialists, such as pygmy-owls (*Glaucidium* spp.) are also diurnal and appear hunt visually (Marks et al. 1999). There is no current evidence to suggest that hearing abilities of avian prey has selected for silent flight in owls.

#### Mammal hearing

Among mammals, owls predominantly eat species that are small (<100 g), including rodents, insectivores, lagomorphs, tenrecs, and small marsupials (Thorstrom et al. 1997; Bruce 1999; Marks et al. 1999; Currie et al. 2003). Virtually all mammals hear, and rodent hearing is well-studied. Most rodents are sensitive to both ultrasonic (>20 kHz) and sonic frequencies. For example, the house mouse (*Mus musculus*) and Norway rat (*Rattus norvegicus*) have an audible frequency range of 5–60 and 1–59 kHz, respectively. Most rodents don’t hear all that well <1 kHz (Ralls 1967; Echteler et al. 1994), but there is an exception that is highly relevant.

The exception are rodents that live in deserts. At least three rodent lineages, kangaroo rats (Heteromyidae), jerboas (Dipodidae), and gerbils (Muridae) have convergently evolved sensitive low frequency hearing (Webster and Plassmann 1992; Mason 2016). Kangaroo rats (*Dipodomys* spp.) have an audible frequency range of 0.1–25 kHz and have been specifically shown to use sound to avoid the predatory strikes of owls (Webster 1962; Longland and Price 1991; Echteler et al. 1994). Experimentally deafened kangaroo rats were less likely than controls to evade owl strikes when there was also insufficient light to see the owl approach (Webster 1962). Thus, there is direct evidence that owls produce enough sound during an attack for a kangaroo rat to hear and evade them. This sound potentially includes low frequency sound. Owls are also a main predator of the other desert rodent lineages (e.g., jerboas, gerbils) that have independently evolved low-frequency hearing (Lay 1974; Leonardi and Dell’Arte 2006; Shao and Liu 2008). Although there are other hypotheses as to why low-frequency hearing has convergently evolved in desert mammals, such as long-distance communication (reviewed in Mason 2016), one viable hypothesis is that sensitive low frequency hearing has evolved specifically for use in defense against predators such as owls (Webster and Plassmann 1992).

### Owl flight sounds (dB_owl_)

Owls hunt for prey and first see or hear prey in two primary contexts: they were either already flying, or they were initially perched. Species such as long-eared owl (*Asio otus*) primarily hunt on the wing using a behavior called coursing (or quartering) (Clark 1975), in which they fly slowly, back and forth low over open areas such as grassland, detecting their prey on the wing at relatively close range (Glue and Hammond 1974). Thus, coursing species make wing noise before prey is first detected or localized but may also detect prey at relatively short distances. Certain diurnal raptors such as harriers that have independently evolved coursing over thick vegetation as a hunting strategy also locate prey by ear (Rice 1982).

Many other owl species are sit-and-wait predators, sitting on a perch above a foraging area, waiting for prey to appear (Peeters 2007). After prey is detected, which may be at a substantial distance, they take off and fly toward the prey. Species employing this strategy only produce wing noises after the attack is launched, since they are sitting still when they first detect prey. Barn owls attracted by a noise may land in the vicinity of the prey, and wait for the prey to make additional sound (Payne 1971a). Upon hearing a sound, they leap into the air, then flap a couple times before striking down from above (Usherwood et al. 2014). Similarly, species hunting prey that are under a layer of snow, such as a Great Gray owl hunting voles, may briefly hover in place while listening for additional prey sounds with which to better localize the meal (Duncan 2003).

Owls finish an attack by thrusting their legs forward to grasp at the prey with their talons (Usherwood et al. 2014), which we hereafter call the “strike.” This strike is performed through a layer of snow, grass, branches, water, or other obstruction as necessary, depending on where the prey is, often producing a loud, sudden crashing or crunching sound as the outstretched talons, and sometimes, various parts of the owl (e.g., wings, tail) strike the substrate. The wing kinematics of a strike on prey have not been described in detail. Extending the talons ahead of the body appears to be accompanied by a pitch-up body rotation. It seems likely that the strike involves wing kinematics and aerodynamic forces that are different than those produced during wing flaps earlier in the attack (i.e., during approach). For example, the wings could be near or at stall during the strike. Thus, the aeroacoustic regime of the wings during the final portion of an attack could differ relative to earlier in the attack, and the wings could reach high angles of attack not experienced during most types of flight.

Payne (1971) found that Barn Owls can hunt in complete darkness, relying only on acoustic cues to accurately locate prey. Their sensitive hearing allows them to localize sounds within 1 degree of accuracy in azimuth from a perch (Konishi 1973b; Rice 1982). Owls can detect changes in the mouse’s trajectory and adjust mid-flight using auditory cues (Payne 1971b). However, experimentation suggests limits to this ability (Konishi 1973a; Hausmann et al. 2008; Ilany and Eilam 2008). Ilany and Eilam (2008) found that Spiny Mice (*Acomys cahirinus*) are more successful at evading the strike when they suddenly fled when a Barn Owl was within 1 m. Mice that fled in the direction of the oncoming owl were the most successful at escaping (Ilany and Eilam 2008). Hausmann et al. (2008) tested course correction accuracy in free flying Barn Owls. The authors played a sound in a new location after takeoff and under certain conditions, the owl navigated to the correct location (Hausmann et al. 2008). In sum, after launching an attack, owls increase their hunting accuracy by continuing to listen for and use prey cues to reorient their attack mid-flight. Thus, self-masking can occur at any point after the attack is launched.

Are owls actually silent? While the flight sounds (dB_owl_) of an owl are not entirely silent, their flight sounds are reduced in comparison to certain other birds. Several studies have recorded the flight noise of live owls, including in anechoic and semi-anechoic conditions (Gruschka et al. 1971; Kroeger et al. 1972; Neuhaus et al. 1973; Chen et al. 2012; Boonman et al. 2018) and in flyover experiments conducted outdoors (Sarradj et al. 2011). These studies all suggest that most species of owls fly relatively quietly (reviewed in Wagner et al. 2017). Thorpe and Griffin (1962) document that owls don’t produce ultrasound in flight, while other birds do.

More difficult than simply documenting the acoustic flight signature is determining what the acoustic mechanisms are that generate the flight sounds of owls and other flying animals. A common experimental paradigm is testing a dried wing in a wind tunnel to mimic gliding (e.g., Geyer et al. 2017). This allows testing of specific aspects of static mechanisms in detail, such as measurement of flow fields over the wing (Geyer et al. 2017) or use of acoustic beamforming to isolate specific sound source locations (Geyer et al. 2013, 2017). However, this only provides limited inference as to which physical mechanisms produce wing sound of gliding. Direct experimental manipulations of wing features hypothesized to affect sound can provide more direct inference into mechanism. Most such experimental manipulations have been of the comb (section “The leading-edge comb”): Some authors have experimentally manipulated the comb on live birds to test what effect it has on flight (Gruschka et al. 1971; Neuhaus et al. 1973). Geyer et al. (2017) experimentally removed the comb from dried wings to test the effect it has on the sound of gliding. By contrast, relatively few studies that have attempted to manipulate the vane fringe and none have manipulated the velvet dorsal surface on a live owl and then recorded the effects of this manipulation on the sounds produce, a point we return to in section “Aerodynamic noise hypotheses.”

#### Sounds of flapping

Tests of dried wings in a wind tunnel have the benefit of being experimentally tractable (Geyer et al. 2013, 2017). However, these tests do not directly address any aspect of sound production that is produced specifically by flapping the wings. Flapping flight has at least two major differences from gliding that are likely to affect sound production. The first is that flapping intrinsically includes changes in the geometry of the wing. Changes in geometry cause feathers to rub against other feathers. The primary way feathers rub is presumably through active (muscle-driven) morphing of the wings (Lentink et al. 2007). Figure 20 in Wolf and Konrath (2015) shows the difference in shape of the wing in upstroke versus downstroke of a Barn Owl; the secondary feathers appear to have morphed (and rubbed) to a greater degree than the primaries. Another geometric aspect that theoretically could produce rubbing is the time-varying cycling of aerodynamic forces on the wings. Cycling of aerodynamic forces cause the feathers of a wing to flex and bend, which potentially causes feathers to slide back and forth against neighboring feathers as they are loaded and unloaded (Graham 1932). Thus, according to either of these mechanisms, the first way that flapping flight potentially produces sound is by producing cycles of geometric wing shape change, causing feathers to rub (slide) against other feathers. These cycles of rubbing potentially produce frictional noise (Akay 2002). We return to this point in the section “The case for structural noise.”

The second way flapping differs from gliding is the pattern of airflow over the bird’s wing is fundamentally altered by flapping (reviewed in Chin and Lentink 2016). In gliding, the local air velocity over the base of the wing is approximately the same as the local air velocity over the tip of the wing. By contrast, an oscillating (flapping) wing has low, relatively unchanging flow at the base of the wing, and fluctuating, higher average airspeeds at the tip. When the airspeed is highest at the tip, such as mid downstroke, there is a velocity gradient down the wing (c.f. gradient on an owl wing in fig. 25 of Wolf and Konrath 2015). This velocity gradient then induces flow down the span of the wing. The effects of this mid-downstroke spanwise flow have not been studied in owls, but in smaller (lower Reynolds number) animals, can include altering the formation and shedding of vorticity on the wing, such as by stabilizing a leading-edge vortex that remains attached to the wing (Birch and Dickinson 2001; Chin and Lentink 2016). The effects of flapping on the airflow over the surface of an owl wing has not been investigated, although the wake of flapping owl has been characterized (Lawley et al. 2019), so it remains unknown how the spanwise velocity gradient significantly affects the acoustic signature of the wing (especially around the leading-edge comb). Tests of the flow field around a static spread bird wing (e.g., Geyer et al. 2017) therefore may not capture all of the aeroacoustic mechanisms that could be present specifically in flapping (Geyer et al. 2014). To what degree the leading-edge comb interacts with spanwise flow during mid-downstroke remains an open question.

#### Role of flapping in owl hunting

The above argument that flapping a wing potentially produces a different acoustic signature than gliding would be moot if the acoustics of flapping did not matter to owl hunting. Since owls have evolved silencing wing features specifically to aid their ability to hunt, an important question is: when hunting, do owls tend to flap their way toward prey, or do they instead glide? Owls hunting by coursing are often flapping when prey is first detected, since they detect prey unpredictably when the owl is already on the wing. Sit-and-wait hunters are perched above a foraging area when they first detect prey, and in theory could glide down to prey located below.

The limited available data do not suggest owls employing a sit-and-wait hunting strategy preferentially glide rather than flap. Mason et al. (2016) presented prey to Northern Saw-whet Owls (*A.* *acadicus*) in an arena in which the owl could glide down to the prey below. Their supplemental videos show individuals flapping, not gliding, during an attack. Western Screech-owl (*Megascops kennicottii*) perched in backyard in Riverside, CA, usually flapped their wings during attacks on mice on the ground below (personal observation). Payne (1971a) describes Barn Owls gliding to attack if they have available light, but in complete darkness, flapping their wings instead. Barn Owls hunting from a perch on the ground, upon detecting prey, leap and then flap their wings (Usherwood et al. 2014). The available data on the role of flapping in hunting summarized above is scant, and, except for Payne (1971), has not been collected in carefully controlled experiments (e.g., in darkness when the owl does not have available visual cues). Nevertheless, based on these data, gliding during attacks appears to be the exception, not the rule. The sounds of flapping are likely a ubiquitous component of owl hunting. Therefore, hypothesis about how owl wing features suppress sounds of flight must address the sound of flapping *per se*, and not just sounds produced by gliding.

### Environmental attenuation (dB_envt_)

Multiple phenomena are contained within the transmission loss term dB_envt_. The simplest form of transmission loss is the effect of distance: sound levels fall according to the inverse of distance (Larsen and Wahlberg 2017; Wahlberg and Larsen 2017). Thus, wing noises produced when the owl is far from prey are selected to be reduced according to the self-masking hypothesis (since the prey sound is faintest at the furthest distance), while wing noises produced close to the prey should be reduced according to the stealth hypothesis, since at shortest distances the prey is likeliest to hear the owl. Any type of wing noises produced specifically in the final stages of an attack, when distance is short (e.g., wing noises caused by the wings at a high angle of attack) may be selected for reduction under the stealth hypothesis, whereas wing noises produced specifically at the onset of an attack (such as spreading the wings to take off) may be selected for reduction according to the self-masking hypothesis.

Apart from distance, the dB_envt_ term includes other forms of environmental attenuation, such as refraction and other scattering caused by temperature gradients, humidity, or wind (including wind gradients and turbulence) in the air between owl and prey (Wahlberg and Larsen 2017). Temperature gradients and wind seem the likeliest, in theory, to affect owl hunting. For instance, moderate winds might carry prey sounds downwind. Shortly after sunset owls may have the advantage of a temperature lapse, in which warmer air by the ground causes sound to refract up, away from the ground, making it easier for the owl to hear prey while reducing owl sounds transmitted to the prey. Temperature inversions (coolest air by the ground, warmer air above) would have the opposite effect. Over the course of nighttime, as the ground cools and winds tend to subside, dB_bkgd_ may reduce and dB_envt_ changes (Wahlberg and Larsen 2017). These forms of environmental attenuation mainly affect sound propagation at distances >10 m. The distances at which owls detect prey has not been documented in detail, so it is unclear to what degree these effects may be ecologically relevant to owl hunting.

**Fig. 3:**
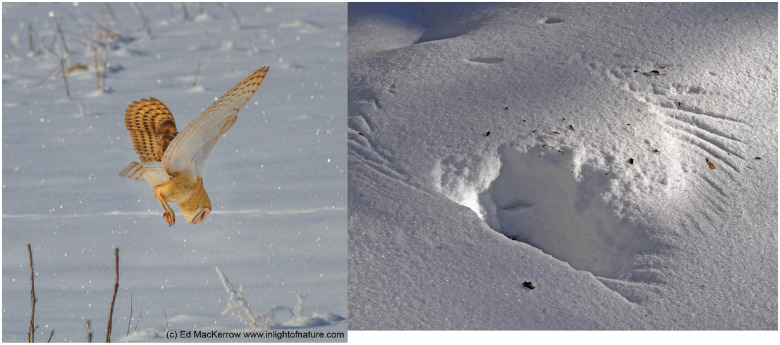
Certain owls hunt through a layer of snow using exclusively acoustic cues to catch prey (e.g., *Microtus* spp.). Left: Barn Owl hunting naturally, courtesy Ed MacKerrow (inlightofnature.com). Right: Barred Owl hunting imprint, courtesy Lee Kensinger.

Another form of environmental attenuation with clear ecological relevance are sounds transmitted through environmental features that attenuate and/or refract the sound, producing large transmission losses. The air–water interface is the most obvious example of this, but both hypotheses predict fishing owls lack silent flight, because dB_envt_ of the air–water is sufficiently large—fish do not hear an approaching owl and an owl does not hear a fish. Snow is less dense than water and provides a more interesting case (Fig. 3). Snow both attenuates sound, and refracts sound as a function of its density (fig. 1 in Capelli et al. 2016), which varies with weather (Judson and Doesken 2000). As snow is a visual barrier, owls hunting prey through snow do so using exclusively acoustic cues. Thus, the two hypotheses make opposite predictions about owls such as Great Gray (*S.* *nebulosa*) or Snowy owl (*B.* *scandiacus*) that hunt prey through a layer of snow. The stealth hypothesis predicts owls hunting through snow will be less likely to have silencing features, since relatively little sound from an attacking owl would reach a rodent even if the owl were not silenced. In fact, when snow is dense, it has a higher speed of sound than air (Capelli et al. 2016), hence sound refracts. Under these conditions, an owl approaching at a shallow angle relative to the snow may have dB_owl_ reflect off of the snow (following Snell’s law), preventing dB_owl_ from reaching the prey under the snow. By contrast, the self-masking hypothesis predicts that owls hunting through snow should have among the best developed silencing features, since relatively little sound from the prey will reach the owl through the snow, yet the owl is completely reliant on sound for hunting, and the background sound is also especially quiet (Fig. 3).

### Background sound (dB_bkgd_)

There are many causes of high levels of background sound, such as rushing water, anthropogenic noises such as roadway noise (Mason et al. 2016), biological sounds such as calling frogs or insects, or wind, which tends to be more prevalent early in the evening before the nocturnal boundary layer has settled in (Garratt 1994). According to both models, production of dB_owl_ matters less to an owl in noisy environments, because high levels of background sound mask the owl’s hearing more so than dB_owl_, and likewise prevent the prey from hearing the owl. A variety of conditions seem likely to present low levels background sound: late at night, because there is less wind (i.e., after the nocturnal boundary layer has fully settled in); in cold environments (reducing singing of nocturnal ectotherms); in snowy environments because snow absorbs sound (Capelli et al. 2016), and far from anthropogenic noises (Mason et al. 2016). Therefore, both models predict that owls tending to hunt in quiet environments should benefit from silent flight.

Among the variables in the two models, dB_bkgd_ is perhaps the easiest to manipulate experimentally. According to the self-masking hypothesis, increases in dB_bkgd_ should reduce owl hunting success, by impairing the owl’s ability to localize prey, whereas according to the stealth hypothesis, increased dB_bkgd_ should increase owl hunting success by instead impairing the prey’s ability to take evasive action in response to owl wing sounds. Mason et al. (2016) showed that experimentally increased dB_bkgd_ reduced the ability of Northern Saw-whet Owls to capture domestic mice (*M. musculus*). These prey may not have been as wary as wild mice (e.g., *Peromyscus* spp.; J. Barber, personal communication), thus their data do not permit evaluation of the stealth hypothesis. Similarly, Senzaki et al. (2016) demonstrated that wild owls have greater difficulty localizing simulated prey sounds at higher levels of background sound. Their methods did not test whether prey, once detected, are made easier to catch by the elevated background sound, as predicted by the stealth hypothesis. Some ecological studies found a negative correlation between owl diversity and abundance in habitats with high levels of anthropogenic sound (Fröhlich and Ciach 2018, 2019), implying owls avoid noisy environments, as predicted by the self-masking model but not the stealth model; but others have not found this pattern (Shonfield and Bayne 2017).

### Which hypothesis is better supported?

These two models are not mutually exclusive and make many predictions that overlap. Both models explain the observation that fishing owls have lost silent flight (Graham 1934), because dB_envt_ of the air–water interface is large. Moreover, dB_bkgd_ is in the denominator of both models. To the degree that certain environments are quieter than others, such as a warm tropical forest (with the incessant nocturnal noises of singing frogs and insects [Waser and Waser 1977]), versus a cold desert, both models predict silent flight in desert specialists over the forest specialists. Since these two hypotheses are not mutually exclusive, the same predator–prey interaction might provide support for both, and there may not ever be definitive support for one hypothesis over the other. Rather, the purpose of generating these hypotheses is to make explicit the assumptions about the underlying mechanisms within the predator–prey interaction that have selected for silent flight, and search for predictions that do differ.

The most obvious difference is that the animal whose hearing ability matters, differs: *k*_owl_ is a parameter of the self-masking hypothesis and not the stealth hypothesis. Owls hunting by ear are predicted to evolve silence according to the self-masking hypothesis. By contrast, *k*_prey_ is a parameter of the stealth hypothesis. Since all owls eat multiple species of prey, *k*_prey_ is an abstraction of the “typical” prey of a species. Owls hunting prey with good *k*_prey_, such as rodents and insects, are predicted to have silencing features, according to the stealth hypothesis.

The second major difference is the role of wing noise (dB_owl_). It is a component of the masker in the self-masking hypothesis, but dB_owl_ is the signal of interest to the prey according to the stealth hypothesis. This means that the role of environmental attenuation is different in the two models: high environmental attenuation (dB_envt_) selects for silencing features under to the self-masking hypothesis, because high attenuation makes prey sounds fainter or harder for the owl to hear, and thus more easily masked by wing noise. By contrast, according to the stealth hypothesis, since high environmental attenuation degrades the prey’s ability to hear wing noise it lowers the need for silencing features. Additional stealth is less needed when the environment already provides stealth naturally.

Finally, the models predict opposite effects of experimentally manipulated levels of dB_bkgd_: increases in dB_bkgd_ should impair owl hunting ability according to the self-masking hypothesis (prey are harder to find), while it should increase hunting ability under the stealth hypothesis (prey have a harder time taking appropriate evasive action). Experiments examining background sound have supported the self-masking hypothesis (Mason et al. 2016; Senzaki et al. 2016), but without simultaneously evaluating the stealth hypothesis. A more comprehensive test of the two models against each other would be to have live owls attack wary prey under variable levels of background sound, similar to the remarkably comprehensive experiments of Longland and Price (1991), who had owls attack wary rodent prey under varying levels of shrub cover and moonlight. An experiment similar to theirs except that simultaneously assessed the effect of dB_bkgd_ on both *k*_owl_ and *k*_prey_ would more directly evaluate the stealth and self-masking hypotheses against each other.

Our interpretation of the limited available data, summarized in Table 2, suggests slightly better support for the masking hypothesis over the stealth hypothesis. The best evidence in favor the owl ear hypothesis is the observation that adaptations for hearing (e.g., the facial disc of *Strix* spp.) seem to be correlated with silencing features; owls hunting through snow also tend to have well-developed silencing features; and increases in dB_bkgd_ cause decreases in detection of prey-like sounds or capture of unwary prey.

**Table 2:** Predictions of the masking and stealth hypotheses of the evolution of silent flight

Ecological condition	Stealth (“mouse ear”) hypothesis predicts	Masking (“owl ear”) hypothesis	Hypothesis supported
Diet	Silent flight predicted in owls hunting prey with good hearing	Silent flight predicted in owls hunting audible prey	
Mammals	Yes (all frequencies)	Yes	Both
Insects	Yes (ultrasound)	No	Stealth?
Birds	No	No	Both
Hunts through snow	No	Yes	Masking
Hunts fish through air–water interface	No	No	Both
Type of sound suppressed	All sound prey can hear (including ultrasound for insects/rodents)	0–10 kHz, especially 3–10 kHz	Both
Sound type and direction	Far-field sound projected in front of the owl	Near and far field sound projected up towards owl’s ear	Untested
Hunts by ear	Not correlated with silencing features	Correlated with silencing features	Untested
Kinematics of attack	Wing noises suppressed when close to prey	Wing noises suppressed when far from prey	Untested
Prey respond to owl wing noises	Yes (desert rodents)	No	Stealth

The strongest evidence in favor of the stealth hypothesis are the data implying that kangaroo rats (*Dipodomys* spp.) can avoid owl strikes in darkness when their hearing is intact (Webster 1962) and that voles (*Microtus socialis*) behaviorally respond to barn owl flight, although this response could be to the tactile components of induced flow from the wings, rather than audible sound (Edut and Eilam 2004; Boonman et al. 2018).

## How do owls have silent flight?

**Fig. 4:**
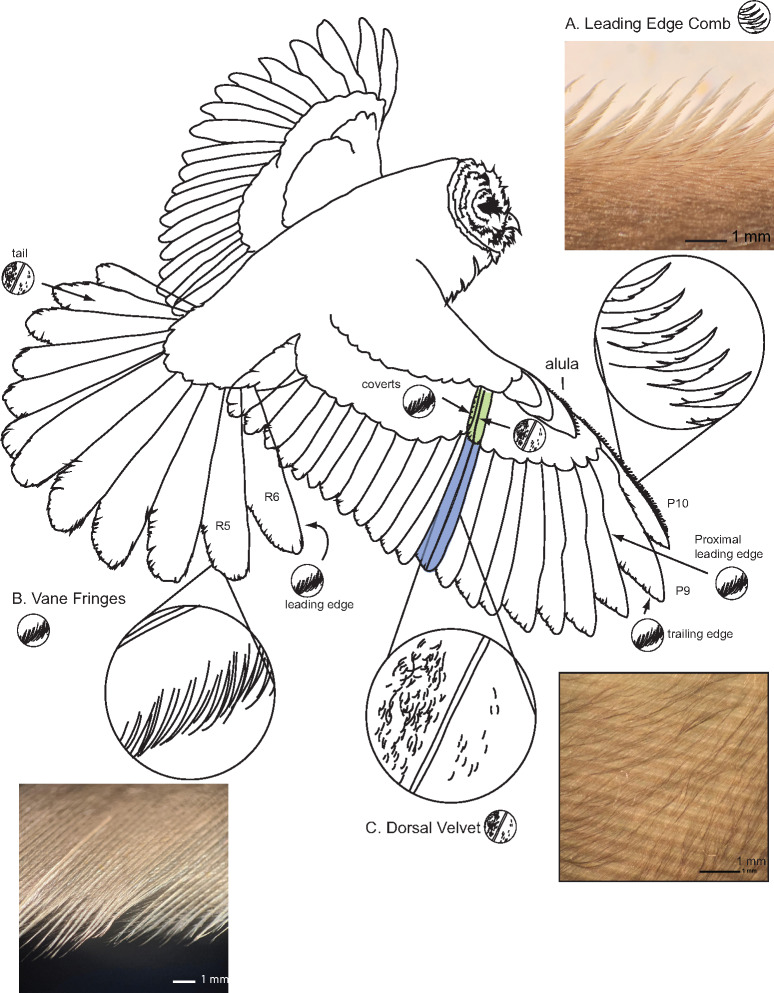
Barred Owl (*Strix varia*) in flight, with **A**) leading-edge comb, **B**) vane fringes, and **C**) dorsal velvet. A) The leading edge comb, in owl species in which it is present, is always on P10, often on the alula, and in certain species, also present on other outer primaries such as P9. B) The vane fringes are present throughout the wings and tail, including the trailing edge of the outer primaries; the leading edge of the primaries (proximal regions, interior of wing); both leading and trailing edges of the coverts (green feather); the leading edge of the tail (where it curves down); and the trailing edge of the tail. C) The dorsal velvet is present throughout the wings and tail, including the remiges, rectrices, and covert feathers. It is longest/most developed on the inner vanes of inner wing feathers (such as P1, in blue). Photos are of Barred Owl feathers, by the authors.

Graham’s (1934) foundational paper identified three morphological aspects of owl wings that were distinct from the wings of most other birds: the leading-edge comb, vane fringes (Graham called this trait the “trailing-edge fringe,” which then became the widespread terminology in the literature), and the velvety dorsal surface of the flight feathers of the wing (Fig. 4). Graham’s “three traits” paradigm is artificial and has channeled subsequent work on this topic. For example, owl feathers also seem to have increased air transmissivity (Müller and Patone 1998) and reduced flexural stiffness (Bachmann et al. 2007) compared with remiges of other similarly-sized birds. This flexural stiffness causes owl wings to bend substantially in airflow (e.g., Winzen et al. 2012; Geyer et al. 2017), perhaps differently from other birds. Moreover, what does and does not constitute a vane fringe has not been precisely defined; is the leading-edge comb simply a type of specialized vane fringe? There seem to be several ways a vane can be fringed (Bachmann et al. 2007, 2012). It will be fruitful for future workers to precisely describe and define the various types of vane fringes that exist across the feathers of owl wings and tails, building on the work of Bachmann et al. (2012). Until such future work is done, we are left with the “three traits” paradigm.

### The leading-edge comb

The leading-edge comb (or serrations) is a row of curved barb tips (Feo et al. 2016) that extend forward from the leading-edge of wing feather P10 and sometimes the leading-edge of the tip of P9, the alula (thumb feathers), and rarely, P8 and P7 (Weger and Wagner 2016; Fig. 4A). The comb of most species projects up (dorsally), meeting the air at the leading-edge of the wing and modifying flow over the dorsal surface of the wing (Rao et al. 2017). The comb on the alula (thumb feathers) on Barred owl projects down (ventrally; *N* = 10 birds), presumably meeting the air at the leading-edge of the wing where it passes under the alula, between the alula and the dorsal surface of the main surface of the wing. In an anatomical sense, the comb may not be entirely distinct from the other vane fringes of the wing (see below). In essence, the comb is a type of vane fringe that differs from other vane fringes in two ways: (1) it has sufficient stiffness to withstand substantial deformation in the presence of aerodynamic forces imposed on it and (2) the proximal barbules on the barbs forming the comb are greatly reduced compared with the distal barbule (see fig. 1 in Weger and Wagner 2016).

Graham (1934) was the first to suggest that the comb reduces aerodynamic noise. Of the three wing features, the comb has received the most study. We only briefly discuss it here, despite its more extensive literature, because possible aerodynamic mechanisms by which the comb affects flow, vorticity, and the ensuing sound were recently reviewed by Wagner et al. (2017); see also Geyer et al. (2017) and Rao et al. (2017), and because we are unaware of any data that dispute the inferred aerodynamic function.

Experiments that manipulated the leading-edge comb on live owls have in some cases yielded little to no change in the sounds of steady, rectilinear flapping flight (Gruschka et al. 1971; Wagner et al. 2017). As the comb is thought to aid in hunting specifically, it is possible that the comb does not affect sound production in all types of flight. Rather, it could have effects specific to aerodynamic regimes or modes of flight (e.g., flapping, gliding) that arise specifically during hunting (section “How owls hunt”). For instance, the comb has been suggested to reduce broadband noise at angles of attack close to stall (Hersh et al. 1974; Geyer et al. 2017; Rao et al. 2017), and/or may delay stall (and the attendant sounds of stall), rather than having an effect at all angles of attack. Removing the comb resulted in an increase in noise just before landing (Neuhaus et al. 1973). These data suggest that the comb may function during the strike (the final portion of an attack), when angle of attack of the wings may be high. Computational fluid dynamics models and empirical measurements suggest the sound suppression provided by the comb is broadband and occurs primarily at frequencies >1 kHz (Rao et al. 2017; Wagner et al. 2017).

In many owl species the comb also curves and twists upward, but there is substantial variation among species in comb morphology with respect to length, inclination angle, and tip displacement angle (Weger and Wagner 2016). Within an individual, comb morphology also varies down the length of the wing. Sick (1937) grouped the leading-edge comb into two categories, the longer, well-defined “Bubo type” with a tapered end and upward curve and the shorter, blunt-tipped “Surina type” (cited in Weger and Wagner 2016). Narrowly defining the comb excludes the shorter morphologies of many species of owl including members of the genera *Athene*, *Bubo*, *Glaucidium*, *Ninox*, *Otus*, *Surina*, and *Tyto*. For example, in *Tyto longimembris*, the barbs on the leading-edge extend straight outward from P10, but don’t curve upward (authors unpublished data). Since these wing features are clearly homologous to the longer (*Bubo* type) combs on other owls, a definition of comb that arbitrarily excludes them is unjustified.

If the definition of the comb includes the short morphologies of many owls, then a comb is not unique to owls: nocturnal frogmouths (Podargidae) have also independently evolved a comb (dashed red lines in Fig. 1B). Their combs are shorter and less curved than the *Bubo* comb type (fig. 30 in Mascha 1905), but overlap with owls of the “Surina” type. Another comb-like wing feature are the short modified barbs extending from the leading-edge of the wings of rough-winged swallows (*Stelgidopteryx* spp.), which are smaller than owls’ combs (fig. 6 in De Jong 1996). The function of these feather modifications in these non-owl species is not known.

### Vane Fringes

Graham (1934) called this feature the “trailing-edge fringe.” We do not use this term because it is inaccurate and misleading to imply this trait is specific to the trailing edge of an owl’s wing. Vane fringes are present throughout the wing and tail of an owl, including on the leading edge of many feathers, and the margin of the vane of covert feathers (Graham 1934; Lucas and Stettenheim 1972; Bachmann et al. 2007, 2012). The majority of vane margins that are fringed are located on feather edges that do not coincide with the trailing edge of the wing (Bachmann et al. 2012). In seeking to draw parallels between owls and aircraft, Graham (1934) applied a name that called attention to this hypothetical source of noise (trailing edge), which was (and is) of considerable interest to aeronautical engineers. To date there have been few empirical tests of trailing edge noise as a source of sound in bird flight (see below). For these reasons, we follow Bachmann et al. (2007, 2012) who carefully described this trait and called this feature the vane fringe.

**Fig. 5:**
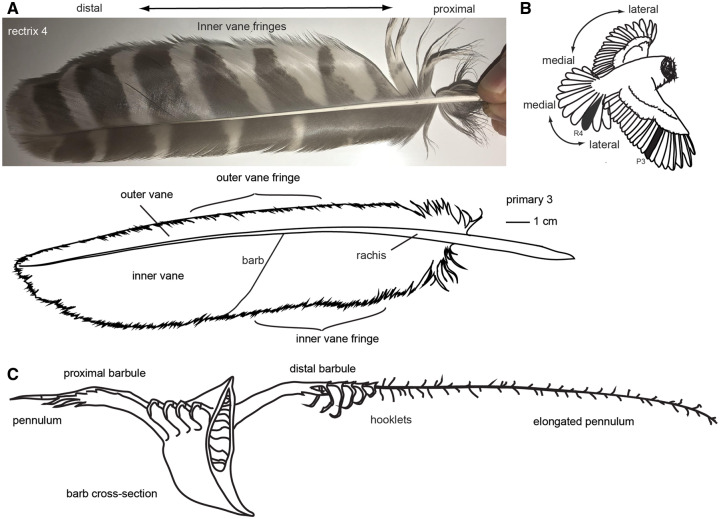
Feather anatomy. **A**) Barred Owl tail feather R4 (photo) and wing feather P3 (line drawing). Feathers are bisected by a shaft (the rachis), and have a vane that is approximately planar, which is made of barbs. The entire vane of many of the rectrices and remiges is fringed, including the inner vane and outer vane; the outer vane fringing curves down to press against the lateral neighboring feather. **B**) lateral–medial axis of wings and tail. Feathers in A are shaded. **C**) Cross section of a barb, showing distal barbule, proximal barbule, hooklets (which attach the distal barbule to neighboring proximal barbule), and elongated pennulum that makes up the dorsal velvet of owl feathers. Drawn following Lucas and Stettenheim (1972).

The region of the feather margin that contributes to the trailing edge of a wing varies with the feather’s position down the wing (Fig. 5A). When a feather’s long axis is perpendicular to flow, which is the case in the outer (more lateral) feathers within the wings and tail, the outer vane is upstream and corresponds to the leading-edge (Fig. 5B), while the inner vane lies downstream (Fig. 5A) and corresponds to trailing edge. By contrast, in relatively medial feathers of both the wings and tail (Fig. 5B), the trailing edge of the wing corresponds to the tip of the feather, while the edges of the inner and outer vane are oriented parallel to flow. Both the outer vanes and inner vanes are fringed in some wing and tail feathers in both Barn Owls (Bachmann et al. 2012) and Barred Owls (*N* = 10), thus we use the terms “outer-vane fringes” and “inner-vane fringes” to refer to fringes in these respective feather regions, irrespective of the direction of airflow over the feather.

A pennaceous feather’s vane is formed by barbs that project off of the shaft of a feather (Fig. 5A). These barbs are hooked to neighboring barbs via barbules (Fig. 5C). Barbules interlock with neighboring barbules with hooklets. This interlocking of barbs via barbules and hooklets forms a flat, continuous surface. The vane fringe is created by the ends of feather barbs, which lack the hooklets that normally anchor a distal barbule to the proximal barbule of the neighboring barb (Bachmann et al. 2007, 2012). Since these hooklets are what hold neighboring barbs together and give the vane a coherent planar structure, their absence causes neighboring barbs to be disconnected and free to move separately at their distal ends. This in aggregate gives the margin of a feather vane a wispy, fringed appearance (Fig. 4B).

Bachmann et al. (2007) documented vane fringes are present on all Barn Owl remiges and wing covert feathers that they investigated, including on the leading vane of some feathers. This fringed region comprises the final 3.5 mm of the inner vane of P10, whereas the fringed region had a length of 1.7 mm on P1 and up to 6.1 mm in a covert feather (Bachmann et al. 2007). That is, vane fringes are *longest* in covert feathers, which are not near the trailing edge of the wing. Bachmann’s result is not unique to Barn Owls (Tytonidae); the wings of Barred Owl also have many primary and secondary feather margins as well as covert feathers are fringed (Figs. 4 and 5A). Also fringed is the leading edge of the tail-feathers including the outer tail-feather, R6 (Fig. 4B;*N* = 10 owls). In addition to owls, some, perhaps many nightbirds have extensive vane fringes in their wing feathers (Mascha 1905); the phylogenetic distribution of this trait within this grade is unclear. We are unaware of any systematic survey of birds to look for this wing feature specifically, so its presence in other bird taxa beyond owls and nightbirds may be overlooked.

### The velvety dorsal surface

The dorsal surface of some parts of the remiges (primary and secondary feathers), alula (thumb feather), upper wing coverts, and rectrices (tail-feathers) is fuzzy. We hereafter use the term “velvet” to describe this structure; “nap” or “pile” are synonyms used by some authors. The term “downy upper surface” (Graham 1934; Kroeger et al. 1972) is another synonym but has occasionally caused confusion (e.g., Lilley 1998), because the term “down feather” also refers to a different type of feather and feather structure, the plumulaceous feathers that lie under the contour feathers of the body of all birds (Lucas and Stettenheim 1972; Gill 2007), i.e., the feathers used to fill down jackets, down blankets, and the like. Downy also refers to plumulaceous barbules found at the base of pennaceous feathers. Down feathers and downy barbules are not known to play a role in sound reduction in flight, and instead serve several other functions, such as insulation (Lucas and Stettenheim 1972). Calling the dorsal surface owl feather feature “velvet” (or nap or pile) avoids this confusion with these two other downy feather structures.

The velvet on owl wing and tail-feathers is hypothesized to be a modified form of friction barbule (Lucas and Stettenheim 1972). Friction barbules are a type of distal barbule modified with a dorsal projection, a cilia, that increases friction with an overlaying medial flight feather when the wing is spread (Proctor and Lynch 1993), where this friction reduces the tendency of these feathers to slip or slide past each other when aerodynamically loaded (Graham 1932; Sick 1937; Oehme 1962; Lucas and Stettenheim 1972). Friction barbules are located on the dorsal surface of the inner vane of the remiges, in the zone of overlap between neighboring remiges (Lucas and Stettenheim 1972).

In owls that have the velvet, the friction barbules are elongated by adding filamentous extension of the pennulum, which may be up to 2 mm long (Lucas and Stettenheim 1972; Fig. 5C). As the density of distal barbules within the vane of a feather is high, the cumulative effect of these elongated pennulae is to cover the dorsal surface of the feather with a nap or pile, giving owl remiges a soft, velvety feel to human fingers. In Barn Owls, the pennulae are longest in regions of flight feathers where they overlap with neighboring flight feathers, such as the inner vane (Bachmann et al. 2007) or proximal portions of outer vane where feathers are prone to rub when the wings are spread or folded (Bachmann et al. 201 their table 2, c.f. their length and density values for 25% versus 75%). The velvet is present, but is relatively short and less developed in wing regions that are directly exposed to the dorsal boundary layer.

The tail-feathers (rectrices) are also covered in the velvet, especially the proximal regions of the feather and on the inner vane (Figs. 4 and 5A) (*N* = 10 Barred Owls). Moreover, owls are not the only species with this feature: some, perhaps many nightbirds also have the velvet in areas of overlap between neighboring remiges (Lucas and Stettenheim 1972), as do Northern Harriers (*Circus hudsoncius*) and White-tailed Kites (*Elanus leucurus*) (Negro et al. 2006; Peeters 2007; Fig. 1). Harriers and kites are both hawks (Accipitriformes), but are not especially closely related, suggesting that the velvet may have evolved independently in each group. We are unaware of any systematic survey for this feature that documents both presence and absence in the wings and tail of a range of species. Its presence in other bird taxa (such as nocturnal species of bird, or other Accipitriformes) may be overlooked.

## Do vane fringes and velvet reduce aerodynamic noise, or structural noise?

We define structural sound as: sounds produced by physical contact (e.g., friction, impact) between a feather and another solid object, especially another wing feather, but also including sounds from incidental collisions with leaves, branches, grass, ground, snow, and other environmental objects. That is, structural sounds are those arising out of interacting solid structures, including frictional and percussive (collision- or impact-based) mechanisms. Graham (1934) hypothesized that the velvety dorsal surface prevented structural noise by allowing feathers to slide past each other noiselessly. This structural noise hypothesis became the accepted explanation for the velvet in the biological literature (e.g., Hertel 1966; Lucas and Stettenheim 1972; Duncan 2003; Peeters 2007). But many papers on owls instead assumed that all of these features have an aerodynamic function by directly interacting with and modifying air as it flows over the wing, thus reducing the acoustic signature of the flow (Kroeger et al. 1972; Lilley 1998).

The logic implicit in Kroeger et al. (1972), Lilly (1998), and subsequent papers is that, since an owl in flight has air flowing over its wing, features that can be found anywhere on wing feathers therefore have evolved to modify the airflow and the ensuing airflow-induced aerodynamic noise sources on the wing. A number have been proposed, including the “velvet boundary layer,” “feather porosity,” and “trailing edge” hypotheses, which we sketch below. We refer to these hypotheses collectively as the “aerodynamic noise hypothesis.” Aerodynamic noise is generated by air moving past or through a structure in any way that produces local fluctuations in pressure, such as through production and shedding of turbulence or vortical structures, including fluctuations in lift and drag (Crighton 1991; Howe 2008; Blake 2017). Aerodynamic noises include familiar everyday sounds of the *whoosh* of a ball flying by one’s ear or a pole swung through the air.

### Aerodynamic noise hypotheses

Hypotheses of aerodynamic noise in bird flight have focused on atonal (broad spectrum) sound sources, rather than tonal sources of aerodynamic noise (such as lift, drag, or vortex shedding). Sources of atonal aerodynamic noise on an airfoil include the formation, scattering, and breakdown of turbulence on the structure itself, including scattering of turbulence as it meets the leading edge or uneven flow at the trailing edge of the wing (Crighton 1991; Howe 2008; Blake 2017). Aerodynamic noises produced by these mechanisms are generally low frequency, on account of the turbulent energy cascade, in which larger vortices initially form and are a source of low-frequency noise, then break into a cascading series of smaller, higher-frequency ones. The smaller vortices are less prevalent and produce less sound because higher frequency vortices viscously dissipate more quickly than the large. Thus, aerodynamic sound produced by turbulence formation and dissipation has a power spectrum that declines exponentially with a charicteristic slope (such as -5/3, log/log scale) with increasing frequency (Crighton 1991; Rao et al. 2017).

The velvety dorsal surface is hypothesized to modulate boundary layer noise of the wing, by modifying the turbulent energy cascade (the “velvet boundary layer” hypothesis) or by affecting aerodynamic separation. Experiments intended to physically replicate the aerodynamic effect of the velvet have placed artificial velvet-like surfaces onto airfoils or other surfaces in wind tunnels (Vad et al. 2006; Klän et al. 2008; Klän et al. 2012; Winzen et al. 2015; Clark et al. 2016; Peake 2016). The underlying measurements from which these artificial surfaces are derived typically come from measurement of the velvet on the inner vane, from the region of overlap with the feather above it (e.g., table 4 in Bachmann et al. 2007), i.e., from velvet on feather regions that are not typically in the boundary layer. The velvet is also hypothesized to make feathers porous in ways that modify the noise produced: the “feather porosity” hypothesis (Kroeger et al. 1972; Geyer et al. 2010, 2012, 2014; Jaworski and Peake 2013). Measurements of air transmissivity show that, at biologically realistic pressures, single owl feathers are somewhat more porous than feathers of other birds (Müller and Patone 1998; Geyer et al. 2014). The next step would be to document how much air passes through an owl’s wing at biologically relevant pressures, especially in wing regions in which multiple remiges and coverts overlap. Whether air transmissivity is affected by the velvet is unclear. Air transmissivity is presumably most affected by the geometry of the entire feather vane, which is structurally dominated by the barbs and barbules. There is not yet any empirical evidence suggesting that the presence of the velvet itself meaningfully changes this porosity, or whether this postulated change in porosity then has a meaningful acoustic effect.

Feather porosity has also been suggested to reduce sound generated by the trailing edge of the wing (Jaworski and Peake 2013)—a version of the “trailing-edge noise” hypothesis. The general version of the trailing edge noise hypothesis states that the morphology of the trailing edge of owl wings (including the trailing edges of splayed individual outer primary feathers) reduces trailing edge noise. For example, the vane fringe might ameliorate sound from scattering caused by shedding of turbulence; or it might reduce the spanwise correlation of shed vorticial structures (reducing their spatial coherence, reducing tonality), or ameliorate other similar violations of the Kutta condition (Kroeger et al. 1972; Lilley 1998; Geyer et al. 2010).

What is missing is empirical evidence that supports any of these proposed mechanisms as the function of the fringed feather vanes or the dorsal velvet. The experimental data documenting that live, gliding owls (Sarradj et al. 2011) or spread wings in wind tunnels (Geyer et al. 2013) are quieter than other birds may be attributable to other mechanisms, such as the leading-edge comb, and/or changes in wing geometry that are an effect of wing flexibility. Studies that have used beamforming to localize sound sources on bird wings have not documented significant amounts of trailing edge noise. Sarradj et al. (2011) used beamforming to measure flight sounds of live birds gliding over a microphone array. Since their birds were moving during measurement, they lacked spatial resolution to be sure sound sources on the wings included trailing edge noise. Subsequent work has not substantiated trailing edge noise as a significant source of sound in bird flight. In a restrained, live pigeon in a wind tunnel, the wingtip (mainly), and the leading edge (to a lesser degree) were the dominant sound sources (Wei et al. 2013 their fig. 3). Dominant sound sources on a hawk (*Buteo buteo*) and Barn Owl wing were localized at points on the wing itself (Geyer et al. 2013 their fig. 6) while in later experiments on a different barn owl wing at both low and high angle of attack (aoa=0° and 24°, respectively), sound sources of unmanipulated wings were predominantly from the middle of the wing (Geyer et al. 2017 their figs. 9 and 10), and not the trailing edge. In total these results are not consistent with the trailing edge as a primary (highest amplitude) source of wing sound, but a stronger test would be to conduct experimental manipulations. There still may be trailing edge noise, it is just not as loud as other sound sources.

**Fig. 6:**
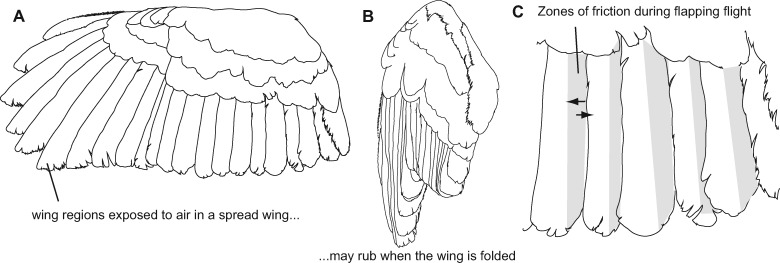
Friction and rubbing within bird wings. **A)** A spread wing has regions exposed to airflow, that **B)** overlap with neighboring feathers when the wing is folded. Thus, a bird spreading its wings to take flight after hearing prey, or tucking them momentarily to maneuver around an obstacle, may generate frictional noises with wing regions that are otherwise exposed to airflow during ordinary flapping or gliding flight. **C)** Secondary wing feathers, showing approximate zones of friction during flapping flight. Line drawings of Barred Owl wing.

Whereas multiple studies have tested the effects of removing the comb (Geyer et al. 2017; Rao et al. 2017), similar studies are lacking for the velvet or vane fringes. To our knowledge, the trailing edge noise hypothesis has been tested only once in live owls: Kroeger et al. (1972) experimentally removed the trailing edge of a Barred Owl’s wing. The result of this manipulation was that flight sounds were reduced, opposite to the result predicted by the aerodynamic noise hypothesis. The effect of the velvet on noise production has never been tested experimentally in an owl, such as by impairing the velvet with hairspray, which is a simple, reversible treatment (Niese and Tobalske 2016). According to the aerodynamic noise hypothesis, physically impairing the velvet should affect the sound of gliding, whereas according to the structural noise hypothesis, it should not, since rubbing is hypothesized to occur primarily during flapping.

#### Evidence against the aerodynamic noise hypothesis

Testable predictions made by the aerodynamic noise and structural noise hypotheses are listed in Table 3 (velvet) and Table 4 (vane fringes). There are five lines of evidence against the aerodynamic noise hypothesis as an explanation for either the velvet or the vane fringes. (1) Neither the velvet nor the fringes are best developed (longest, thickest, densest) in the wing regions where they should be if their function were to modify airflow. (2) Aerodynamic noise hypotheses do not predict the presence of well-developed velvet and vane fringes in owls’ tails. (3) Aerodynamic mechanisms of sound production are inconsistent with the broadband acoustic spectrum that includes substantial ultrasound which is widespread in flapping flight of “regular” bird flight (see the section “Do other flying animals have silent flight?”). (4) Arguments about trailing-edge aerodynamic mechanisms tend to disregard the geometry of the problem owls are “trying” to solve. Neither mouse ears nor owl ears are located behind the owl as it flies (section “Why do owls have silent flights?”). Thus, neither hypothesis about the evolution of silent flight predicts that owls have evolved wing features to reduce noise projected behind the bird *per se*. Any reduction in noise projected behind, above, or below the animal is a correlated byproduct (an exaptation: Gould and Vrba 1982) of a wing feature selected to reduce flight sound projected inwards toward the owl’s ears or forward, toward the prey’s ears.

**Table 3:** Predictions of the aerodynamic and structural noise^a^ hypotheses of the function of the dorsal velvet

Data	Aerodynamic noise hypothesis predicts	Structural noise hypothesis predicts	Data	Hypothesis supported
Anatomical location of velvet	Most developed in aerodynamic surface of wing (areas most exposed to airflow)	Most developed between feathers (in wing regions exposed to rubbing)	Most developed between feathers (Bachmann et al. 2007)	Structural noise
Experimental manipulation of velvet	All wing noise increases (including gliding)	Noises of flapping but not gliding increases	Untested	Untested
Present in tail?	Absent	Present	Present on tail (Fig. 4)	Structural noise
Flight speed	Flapping noise rises with airspeed	Weak or no relationship with airspeed (structural noise may be present at any speed)	Untested	Untested
Rubbing feather against another feather	Velvet does not affect rubbing (frictional) noise	Velvet reduces feather frictional noise	Velvet reduces frictional noise (Lucas and Stettenheim 1972)	Structural noise
Flight sound spectrum	Bird flight sounds are predominantly low frequency	Bird flight sounds are broadband, including ultrasound	Flight sounds broadband (Fig. 7A)	Structural noise^b^
Evolutionary development	Homology with aerodynamic structures	Homology with frictional structures	Pennulae homologous to friction barbules (Lucas and Stettenheim 1972)	Structural noise

^a^Frictional sound produced by the surface of the vane rubbing against other surfaces, such as the neighboring medial feathers.

^b^Evidence is not be specific to the velvet (Table 4).

**Table 4:** Predictions made by the aerodynamic and structural noise^a^ hypotheses of the vane fringe functions^b^

Data	Aerodynamic noise hypothesis predicts	Structural noise hypothesis predicts	Data	Hypothesis supported
Anatomical location of fringe	Present only on trailing edge of wing	Present on margins of feathers anywhere within wing, including leading-edges	Present anywhere in wing including leading-edges (Bachmann et al. 2012)	Structural noise
Experimental removal of trailing edge	Wing noise increased	No effect	Sound reduced (Geyer et al. 2014)	Structural noise
Present in tail?	Absent	Present	Present on tail (Fig. 4)	Structural noise
Flight speed	Noise rises with a high exponent of airspeed	Present at any speed	Untested	Untested
Rubbing feather against a substrate	Fringing does not affect frictional sounds	Vane fringes reduced frictional noise	Untested	Untested
Flight sound spectrum	Bird flight sounds are low frequency	Bird flight sounds are broadband, including ultrasound	Broadband sound present (Fig. 7A)	Structural noise^c^

^a^Structural noise of the vane margin is produced by the feather edge rubbing against other feathers or other environmental objects.

^b^Often termed the “trailing edge fringe,” but is not restricted to the trailing edge (see the text).

^c^Evidence is not specific to the vane fringes (Table 3).

(5) Sources of aerodynamic sound generally scale at a high exponent of airspeed (U), such as U^4^, U^5^, or U^6^ (Crighton 1991; Lilley 1998; Geyer et al. 2010). Owls generally fly slowly, at speeds of <10 m s^−1^ (Longland and Price 1991; Wolf and Konrath 2015). A process that produces a 100 dB sound at 100 m s^−1^ and scales as U^5^, as trailing edge noise might (Lilley 1998), will at 10 m s^−1^ produce 0 dB of sound. Some aerodynamic mechanisms of sound production relevant at the high speeds aircraft fly may be trivial at the low speeds owls fly. For instance, Clark et al. (2016, their fig. 16c) found a velvet-inspired canopy attenuated radiated sound by up to 5 dB at an airspeed of 21 m s^−1^ but essentially no difference an airspeed of 10.1 m s^−1^, similar to the highest speeds owls fly.

If an animal is selected to reduce the sounds of flight during hunting, then, all else equal, it is selected to reduce the loudest sources of wing-generated sounds. Thus, the starting point for understanding how owls reduce their flight noise is to ask: what is the loudest source of wing noise in a flying bird? As shown above, owls flap their wings while hunting (section “Role of flapping in owl hunting”) which means their acoustic signature potentially includes the effect of individual feathers rubbing against each other, particularly in the secondaries (section “Sounds of flapping”). The limited available evidence, presented next, suggests that both the vane fringe and velvety dorsal surface reduce structural noise, such as caused by rubbing between neighboring feathers (Graham 1934).

### The case for structural noise

Bird wings are made from many individual feathers. As individual feathers are separate and semiautonomous, they may slide past each other. Three flight regimes in which sliding is especially likely are: flapping flight, in which the wing morphs and feathers flex and deform periodically under aerodynamic load (Graham 1932; Wissa et al. 2015; Wolf and Konrath 2015); when a perched owl takes off, spreading its wings from an initially folded position (Bachmann et al. 2007, 2012); and when an owl tucks its wings to maneuver, such as when flying through a gap in vegetation, or in the strike. Why does the tail also have the same silencing features as the wings? The structural noise hypothesis predicts the tail feathers could make frictional sound when the tail is spread during a maneuver.

#### Frictional sound characteristics

What type of sound is predicted by friction? Two solid structures sliding past each other can generate a variety of acoustic forms, depending on the loading regime and geometry of the structures (Akay 2002). Feathers are rough, and contain many small asperities, the barbs and barbules, at a sub-millimeter scale. If two rubbing structures each have many small asperities (such as two pieces of sandpaper), when sheared (slid against each other), these small asperities have many local, semi-independent interactions with asperities on the opposing surface. If these asperities are non-uniform in geometry and loading, and so have a broad distribution of excited frequencies, the structural noise from rubbing is predicted to be broadband (atonal) (Akay 2002).

Frictional noise is therefore predicted to substantially differ from aerodynamic noise. Unlike turbulence noise predicted by the aerodynamic noise hypothesis, the power spectrum of frictional noise is not expected drop exponentially with increasing frequencies. It is expected to include substantial high frequency sound, including ultrasound (Akay 2002), and could have a flat power spectrum. Moreover, unlike aerodynamic noise, structural noise is not expected to scale with a high exponent of flight speed of the animal. The feathers may rub as the wings are flapped even when the bird flies slowly, thus wing noises might scale weakly or not at all with U, rather than with a high exponent of U as predicted by the aerodynamic noise hypothesis.

#### Sound and collisions

Our definition of structural noise also includes the sounds of impacts. We hypothesize that nocturnal animals occasionally collide with environmental objects as they fly (section “How owls hunt”), since many species fly in the dark, when visual feedback about the location of environmental objects, such as branches, is limited. A hunting owl may graze a bush as it flies past, then moments later, crash through a layer of dry grass as it strikes at prey hidden underneath. According to this hypothesis, nocturnal animals may be under selection to evolve wings that are more resistant to collision-induced sound (and damage) than are diurnal flyers. Thus, the structural noise hypothesis predicts that owl wing features may also reduce the sound of these collisions with environmental objects, in addition to reducing the sound of rubbing.

### Velvety dorsal surface and frictional noise

The velvety surface not only magnifies the friction between the feathers but also quiets the sound of the feathers rubbing against each other

    (Lucas and Stettenheim 1972, pp. 261).

The velvet may serve to reduce frictional noise generated by contact between the surface area of one feather against the surface area of another feather, one broad flat surface loaded in shear against another broad surface (Fig. 6). Therefore, according to this frictional noise hypothesis, the velvet is predicted to be best developed in regions where feathers overlap with other feathers (Table 3). In Barn Owls, this is precisely where the velvet is longest and densest, including the proximal region of the primary feathers, in the proximal regions of secondary feathers, and especially, underneath the upper wing covert feathers (Fig. 4) (Bachmann et al. 2007). Therefore, owl wing anatomy is consistent with the hypothesis that the primary function of the velvety dorsal surface is to reduce the sound of feathers sliding against feathers (Bachmann et al. 2011), and not consistent with the hypothesis that the velvet affects the boundary layer.

The velvet in owls is present, in reduced form, across the entire aerodynamic surface of Barn Owl flight feathers, including in regions of wing feathers that are exposed to airflow throughout the entire wingbeat cycle of a wing during flapping flight. These regions do not experience cycles of a neighboring feather rubbing over the top of them during flapping (Fig. 6). These feather regions include the entire tips of the outer primaries (e.g., p9 and p10) and the vane near the leading-edges of all wing feathers (Bachmann et al. 2011). This velvet on these wing regions is in airflow during flapping, and could plausibly have evolved to modify the boundary layer. Alternately, this anatomy can still be explained by the structural noise hypothesis. The wings are not always fully spread during hunting (Fig. 6A; section “Owl flight sounds (dB_owl_)”). A perched owl holds its wings folded; thus, these exposed areas potentially rub against other feathers when the wing is first spread. Birds of all sorts, including owls, tuck their wings when maneuvering through a narrow gap in vegetation, and also tuck or fold their wings in the strike on prey (Konishi 1973a). Any part of a wing may collide with environmental objects in the cluttered environments in which owls often hunt. According to this hypothesis, the velvety dorsal surface that is exposed to air when the wing is flapped may nevertheless function to reduce structural sounds when that part of the wing physically touches another structure.

According to the aerodynamic noise hypothesis, the velvet’s function is to modify the boundary layer over the wing. This hypothesis does not predict its presence in the tail feathers. The tail does not experience the same flow conditions as the wings; the tail is not thought to have a thin boundary layer, for instance, because it lies in the wake of the body (Maybury and Rayner 2001; Maybury et al. 2001). Thus, the aerodynamic noise hypothesis does not predict it would have the same silencing features as the wings. The velvet is well-developed in owl tail feathers (Fig. 3A), especially in the inner vane, where the feather overlaps with its proximal neighboring feather, broad surface area of one flight feather against broad surface area of the adjacent flight feather. The structural noise hypothesis predicts its presence there, as the tail-feathers may be just as prone to rubbing and collisions as are the wing feathers, when the tail is spread during a maneuver.

**Fig. 7:**
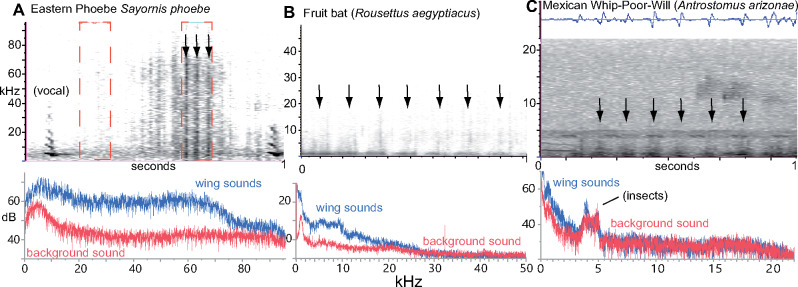
Wing sounds of a **A**) passerine, **B**) megachiropteran bat, and **C**) nightbird (Caprimulgidae). Graph A shows broadband wing sound up to 70 kHz; Graphs B and C show reduced broadband sound and virtually no ultrasound. Vertical arrows indicate individual wingbeats. Bottom power spectrum of wing sounds (blue) and background sound from the same recording (selected as in red dashed boxes in A). A) Eastern Phoebe flight sound recording courtesy Jayne Yack (Fournier et al. 2013) (FFT settings: 200 kHz, Hann, 4096 sample window, see Fournier et al. 2013 for dB calibration), recorded <0.5 m from the animal. B) Egyptian Fruit Bat (*Rousettus aegyptiacus:* Megachiroptera), recording courtesy Yossi Yovel (Boonman et al. 2014), from a microphone attached to the animal (FFT settings: 100 kHz, Hann, 2048 sample window, dB not calibrated). C) Mexican Whip-Poor-Will (*Antrostomus arizonae*) taking flight near a nest, recorded by a microphone placed approx. 1 m from nest. Top: individual wing-beats are clearly visible in wave-form. Recording courtesy Nathan Pieplow; 44.1 kHz, 512 sample window, dB not calibrated.

The structural noise hypothesis makes a series of additional predictions that are each supported by the available data. According to the structural noise hypothesis, birds lacking the velvet or vane fringes should produce flight noises that are broadband and includes ultrasound (>20 kHz), since this is the acoustic signature of rubbing (and not aerodynamic noise). The data support this prediction. Thorpe and Griffin (1962) recorded ultrasound in bird flight using a bat detector, and report that most owls lacked ultrasound in flight, whereas some pigeons, a falcon, and a hawk all produced ultrasound, as did fishing owls that lack the silencing wing features (*Bubo ketupu* and *Bubo ussheri*) (Table 5). Spectra of Thorpe and Griffin (1962)’s birds were not available, but they are available for a more recent study. Fournier et al. (2013) measured wing noises of two oscine passerines, Eastern Phoebe (*Sayornis phoebe*) and Black-capped Chickadee (*Poecile atricapillus*). Their recordings show that the wing noises of these species include ultrasound up to about 70 kHz (Fig. 7A and Table 4). This nearly flat power spectrum (slope of 0 up to about 60 kHz) is consistent with a frictional noise source mechanism, and not consistent with the aerodynamic noise hypothesis, which predicts a steep decline at higher frequencies (such as a slope predicted by the turbulent energy cascade of −5/3).

**Table 5:** Reports of ultrasound in vertebrate flight

Species	Method	Ultrasound?	Reference
*Otus scops*	Bat detector	Absent	Thorpe and Griffin (1962)
*Athene noctua*	Bat detector	Absent	Thorpe and Griffin (1962)
*Strix aluco*	Bat detector	Absent	Thorpe and Griffin (1962)
*Tyto alba*	Bat detector	Absent	Thorpe and Griffin (1962)
*Asio otus*	Bat detector	Absent	Thorpe and Griffin (1962)
*Pseudoscops* (*Rhinoptynx*) *clamator*	Bat detector	Trace ultrasound on takeoff	Thorpe and Griffin (1962)
*Strix seloputo*	Bat detector	Almost silent	Thorpe and Griffin (1962)
*Bubo bubo*	Bat detector	Quiet but not silent	Thorpe and Griffin (1962)
*Bubo virginianus*	Bat detector	Quiet but not silent	Thorpe and Griffin (1962)
*Ketupa* (*Bubo*) *ketupu*	Bat detector	Present	Thorpe and Griffin (1962)
*Scotopelia (Bubo) ussheri*	Bat detector	Present	Thorpe and Griffin (1962)
*Falco tinnunculus*	Bat detector	Present	Thorpe and Griffin (1962)
*Accipiter badius*	Bat detector	Present	Thorpe and Griffin (1962)
*Columba livia*	Bat detector	Present	Thorpe and Griffin (1962)
*C. palumbus*	Bat detector	Present	Thorpe and Griffin (1962)
*Streptopelia turtur*	Bat detector	Present	Thorpe and Griffin (1962)
*S. risoria*	Bat detector	Present	Thorpe and Griffin (1962)
*Spilopelia senegalensis*	Bat detector	Present	Thorpe and Griffin (1962)
*Spilopelia chinensis*	Bat detector	Present	Thorpe and Griffin (1962)
*Sayornis phoebe*	100 kHz microphone	Present	Fournier et al. (2013; Fig. 7A)
*Poecile atricappilus*	100 kHz microphone	Present	Fournier et al. (2013)
*Rousettus aegyptiacus*	50 kHz microphone	Absent	Y. Yovel (personal communication; Fig. 7B)

The structural noise hypothesis also predicts that, in regular birds, flapping flight is noisier than gliding, because rubbing present in flapping should be largely absent in gliding. Although this has not been formally tested, everyday observations of wild birds in flight suggest this hypothesis is supported. Wing flapping of many birds are distinctly audible events, which in many species seem louder than gliding. However this observation is not universally true: some species, such as vultures or hornbills also produce substantial sound in gliding (Clark and Prum 2015), suggesting that a quantitative and phylogenetic analysis of this trait is warranted.

The structural noise hypothesis also has support from an evolutionary development perspective. Lucas and Stettenheim (1972) suggest that friction barbules are widespread in the wings of birds, in zones of overlap between neighboring feathers (Proctor and Lynch 1993, pg. 85). The postulated function of friction barbules is to increase friction between neighboring feathers, preventing or controlling slipping under applied shear forces that develop between adjacent feathers when the wing produces aerodynamic forces (Wissa et al. 2015). Lucas and Stettenheim (1972) hypothesize that the pennulae on owl wings are homologous to and simply a modified form of friction barbule. Frictional sounds are most likely to be produced, first and foremost, in the specific regions of friction between feathers, where the feathers already contain frictional barbules. In fact, friction barbules themselves are expected to be the source of friction sounds, since friction barbules are the asperities, the major point of contact between the two feathers. Thus, frictional barbules are the likeliest structures to be modified if a new selective pressure arose that selected against frictional sounds.

Finally, the structural noise hypothesis makes another prediction. It predicts that bats don’t produce ultrasound in flight, because they lack feathers, and thus do not have feathers that rub against feathers when they fly. This prediction is supported for at least one species: wing noises of one bat species lack ultrasound (Fig. 7B; Y. Yovel, personal communication).

There are no lines of evidence that unambiguously support any of the versions of the aerodynamic noise hypothesis for the velvet (Table 3). The lines of evidence consistent with the aerodynamic noise hypothesis, such as wind tunnel experiments showing that static owl wings are quieter than wings of other birds, can be explained by other features of owl wings, such as the leading-edge comb or the reduced flexural stiffness of owl wing feathers. There are several independent lines of evidence that instead support the structural noise hypothesis with respect to the velvet. The structural sound hypothesis is consistent with the anatomy of the velvet (the velvet is longest in regions where feathers overlap; the velvet is present on tail-feathers). The structural sound hypothesis is consistent with data from live birds: birds lacking the velvet produce broadband noise in flight, while owls and bats don’t produce broadband noise (Fig. 7). The structural sound hypothesis is consistent with observation that wing flapping is a distinctly audible event; and from the apparent homology between the velvet of owls, and friction barbules present in the wings of other birds. Additional tests of this hypothesis could include experimental manipulation of a live bird, for example, this hypothesis predicts that adding a lubricant between the wing feathers reduces sound in a bird with noisy flight or showing that experimentally impairing the velvet (such as with hairspray) of a live owl increases sound during flapping flight but not gliding flight.

#### Vane fringes and frictional noise

Because the trailing edge fringe is present on the rear margins of all the primary feathers, it well might be thought that its purpose has to do with reducing friction between overlapping feathers (Graham 1934, 842).

Graham (1934) proposed that the vane fringes function to ameliorate aerodynamic sound from the trailing edge of the wing, rather than structural sound. As the above quote reveals, he was aware that the anatomical evidence did not unequivocally support his preferred hypothesis, because the fringe extends up each inner vane of the outer primaries, beyond the feather region that corresponds to the trailing edge of the whole wing. He argued that during the upstroke gaps appear between all of the feathers during slow flight, and thus the trailing edge of the wing would include interior portions of the wing during the upstroke. Graham died when Stuka dive-bombers sank the destroyer *Bison* (Anonymous 1940), so he did not get a chance to present his photographic evidence of gaps in the wing during the upstroke.

Modern images of owl wings during the upstroke (e.g., Usherwood et al. 2014) suggest gaps form rarely among the primaries, primarily when owls fly at low speed. Contrary to Graham’s (1934) hypothesis, the distribution of the fringes across the wing are not coincident with the locations of these wing gaps during slow flight. Vane fringes are present in wing locations that are never near the trailing edge of the wing, such as the upper wing covert feathers (Bachmann et al. 2012); in the tail-feathers (*N* = 10 Barred Owls), and that the fringe is longer in parts of primary feathers that *overlap* with neighboring feathers rather than in the free (trailing edge) portion of the feather (Bachmann et al. 2012). The fringe is present on the leading-edge of most wing feathers, where, in Barred Owl, it curves down from the plane of the feather (*N* = 10), as well as the leading-edge of the outer tail-feathers; Fig. 4B (*N* = 10). This anatomical pattern suggests that the function(s) of the vane fringe is not restricted to the trailing edge of the wing, but rather, the function is distributed along the edges of all flight feathers, particularly where the margin of one feather overlaps with their neighboring feathers.

The aerodynamic noise hypothesis, with respect to the fringe, has been tested once in live owls: Kroeger et al. (1972) experimentally removed the trailing edge of a Barred Owl’s wing (data replotted in fig. 3 of Geyer et al. 2014) but found that flight sounds were reduced, rather than increased, as expected, relative to the unmanipulated bird. Kroeger et al.’s (1972) sample size is small, and the precise manipulation they performed is not documented. This experiment bears repeating with a larger sample size of birds and more explicit methods. Kroeger et al.’s (1972) result, if it is replicable, fails to support the aerodynamic noise hypothesis with respect to the vane fringes.

Bachmann et al. (2012) proposed that the fringe serves to reduce friction between two neighboring feathers as they slide past each other. Specifically, the fringes of a feather “merge tightly into the grooves formed by the adjacent barb shafts” of the proximal feather (Bachmann et al. 2012), and prevent friction. This loading regime has an important difference from that of the velvet dorsal surface: whereas the velvet forms a layer on the broad surface areas of a feather (“surface friction”), the vane fringe instead softens the edge of the pennaceous feather, preventing friction sounds caused by the edge of the feather sliding against another structure. This edge-on friction (“edge friction”) includes, but is not limited to, friction with other parts of the owl’s wings such as those described by Bachmann et al. (2012), as well as friction and impact with environmental objects, especially during the strike (section “Vane fringes and frictional noise”).

## Do other flying animals have silent flight?

Of the three wing features described above, a comb has also evolved in frogmouths (*Podargus*; Fig. 1; Mascha 1905), as well as in Rough-winged Swallows (*Stelgidopteryx* spp.). Nothing is known about the function of these traits in these species.

The velvet appears to have evolved at least four times in birds (Fig. 1). As proper absence data have not been collected and this trait is easy to overlook, this number of independent origins will likely change with additional research attention. Harriers have apparently convergently evolved a velvety nap on their secondary wing feathers (Peeters 2007), as has the crepuscular White-tailed Kite (Peeters 2007). The velvet appears to be widespread within at least two of the six lineages within Caprimulgiformes. In nightjars (Caprimulgidae), digital images on featherbase.info, the US Fish and Wildlife Service’s Feather Atlas (www.fws.gov/lab/featheratlas/), and examination of a few specimens at the Museum of Vertebrate Zoology indicates this character is present in one or more species in the nightjar genera *Camprimulgus*, *Phalaenoptilus*, *Eurostapodus*, and *Nyctidromus* suggesting it may be widespread within nightjars. The velvet is also present in frogmouths (Mascha 1905), and absent in Apodiformes. The distribution of this character in other three clades within Caprimulgiformes (*Steatornis*, Aegothelidae, and Nyctibidae) is unknown.

The structural noise hypothesis predicts that the velvet suppresses broadband frictional sound, and thus it predicts that these species do not produce broadband sound during flight. We did not locate any sound recordings of Harriers or Kites that would permit a test of this hypothesis in those taxa. A recording of a Nightbird, a Mexican Whip-poor will (*Antrostomus arizonae*) recorded at close range flying to its nest, shows little sound >3 kHz, supporting the structural noise hypothesis (Fig. 7C).

### Are owls quiet, or is bird flight noisy?

The structural noise hypothesis suggests that feathers make flight noisier, since they are semiautonomous structures that can rub against each other in flight. Thus, the question of silent flight could be re-framed: Is it really owls that are quiet, or instead, is bird flight noisy? There is another clade of nocturnal flying animals that is speciose and fly at similar Reynolds numbers to birds: bats (Chiroptera). Bat wings are made of patagium: an aerodynamic surface of skin, not feathers (Norberg 1990). Because they naturally lack feathers, according to our structural noise hypothesis, bats have not needed to eliminate the sounds that feathers produce as they rub past each other. Studies of silent flight may find it productive to take a broader perspective. Comparing owls to birds with intrinsically noisy flight such as pigeons or ducks does little to reveal just how quiet owls really are. Is bat flight as quiet as owl flight? There are many possible comparisons to make (flapping versus gliding, sound as a function of wing loading, different flight speeds, nocturnal versus diurnal bats, etc.). Like owls, some bat species home in on prey sounds they passively hear while flying overhead (Bell 1982), implying they could be selected for silent flight under the self-masking hypothesis. Have any bats evolved wing features that reduce their flight noises? If any of the postulated aerodynamic mechanisms such as distributed porosity or trailing edge effects do reduce the sound levels produced by owls in flight, then owl flight is predicted to be quieter than bat flight.

## Conclusions

There is still much to learn about how and why vertebrates have evolved silent flight. Here, we have reviewed what is known about the ecological conditions surrounding the evolution of silent flight. The available data are inconsistent with several assumptions about owl flight widespread in the literature. These ideas include: (i) owl flight is stealthy; (ii) owl wing features promoting silence all do so by reducing aerodynamic noise; (iii) certain owl wing features reduce far-field noise radiated behind the owl; (iv) only owls have quiet flight; and (v) owls are the ideal model for bioinspiration of silent flight. The existing data, such as they are, instead suggest: (i) owls may fly quietly to reduce self-masking (but this is uncertain); (ii) a principle function of the vane fringes and velvety dorsal surface may be to reduce structural (frictional and percussive) noise; (iii) owls have not evolved to reduce the sound shed behind them *per se*, because neither the owl’s ears nor prey’s ears are behind the owl; (iv) a few other bird groups that share certain ecological similarities with owls seem to suppress flight sounds as well; and (v) bats lack feathers which may make their flight intrinsically quiet, and thus might be a good comparison with owls.

There are many possible follow-up experiments that appear to be relatively simple to pursue. One of the most obvious omissions in the literature is comparative data on the sounds bats produce as they fly. Study of the flight noises produced by a phylogenetically diverse array of birds and similarly-sized bats, flown under similar (preferably anechoic) conditions, would test whether owls are actually silent, relative to a flyer that intrinsically lacks feathers, or whether it is other birds that are noisy. Comparative phylogenetic analyses with museum specimens also could fairly easily answer some of the other questions raised here. For instance, what are the ecological correlates with the evolution of silent flight, is it nocturnality, or hunting by ear? There might be a clear answer in the distribution of wing features thought to be associated with silent flight within the entire Nightbird grade. Given its interesting phylogenetic placement, use of echolocation, and unique fruit diet, does the Oilbird (*Steatornis caripensis*) have silencing features? The stealth and masking hypotheses of silent flight make certain contrasting predictions about the evolution of owl ears. A phylogenetic analysis of owl wing and ear morphology could test hypotheses about the function and evolution of silent flight. Moreover, the stealth and self-masking hypotheses are not limited to owls, but apply to any predator–prey interaction involving flight, such as nightbirds or bats. Finally, further assessment of how owls hunt in the wild, including basic psychoacoustic questions (how do owls account for possible environmental refraction of prey sound by snow or thermal gradients?) would be fascinating.
